# Proinflammatory NFkB signalling promotes mitochondrial dysfunction in skeletal muscle in response to cellular fuel overloading

**DOI:** 10.1007/s00018-019-03148-8

**Published:** 2019-05-17

**Authors:** Raid B. Nisr, Dinesh S. Shah, Ian G. Ganley, Harinder S. Hundal

**Affiliations:** 1 Division of Cell Signalling and Immunology, Sir James Black Centre, School of Life Sciences, University of Dundee, Dundee, DD1 5EH UK; 2 MRC Protein Phosphorylation and Ubiquitylation Unit, Sir James Black Centre, School of Life Sciences, University of Dundee, Dundee, DD1 5EH UK

**Keywords:** NFkB, Muscle, Glucose, Palmitate, Insulin, Mitochondria, Respiration, Metabolism

## Abstract

Sustained nutrient (fuel) excess, as occurs during obesity and diabetes, has been linked to increased inflammation, impaired mitochondrial homeostasis, lipotoxicity, and insulin resistance in skeletal muscle. Precisely how mitochondrial dysfunction is initiated and whether it contributes to insulin resistance in this tissue remains a poorly resolved issue. Herein, we examine the contribution that an increase in proinflammatory NFkB signalling makes towards regulation of mitochondrial bioenergetics, morphology, and dynamics and its impact upon insulin action in skeletal muscle cells subject to chronic fuel (glucose and palmitate) overloading. We show sustained nutrient excess of L6 myotubes promotes activation of the IKKβ-NFkB pathway (as judged by a six-fold increase in IL-6 mRNA expression; an NFkB target gene) and that this was associated with a marked reduction in mitochondrial respiratory capacity (>50%), a three-fold increase in mitochondrial fragmentation and 2.5-fold increase in mitophagy. Under these circumstances, we also noted a reduction in the mRNA and protein abundance of PGC1α and that of key mitochondrial components (SDHA, ANT-1, UCP3, and MFN2) as well as an increase in cellular ROS and impaired insulin action in myotubes. Strikingly, pharmacological or genetic repression of NFkB activity ameliorated disturbances in mitochondrial respiratory function/morphology, attenuated loss of SDHA, ANT-1, UCP3, and MFN2 and mitigated the increase in ROS and the associated reduction in myotube insulin sensitivity. Our findings indicate that sustained oversupply of metabolic fuel to skeletal muscle cells induces heightened NFkB signalling and that this serves as a critical driver for disturbances in mitochondrial function and morphology, redox status, and insulin signalling.

**Electronic supplementary material:**

The online version of this article (10.1007/s00018-019-03148-8) contains supplementary material, which is available to authorized users.

## Introduction

There is mounting evidence that chronic activation of proinflammatory signalling in tissues such as skeletal muscle and adipose is a significant contributing factor in the development and progression of metabolic disorders such as insulin resistance, obesity and Type II diabetes [1, 2]. This inflammatory response is triggered by circulating proinflammatory cytokines such as interleukin-6 (IL-6), tumour necrosis factor-α (TNFα) and by sustained increases in the concentration of free fatty acids (FAs), such as palmitate. The actions of these stimuli serve to not only further induce tissue expression and secretion of IL-6 and TNFα (via the NFκB pathway, [3, 4]), but impair control of numerous signalling pathways regulating skeletal muscle insulin signalling, glucose uptake, mitochondrial fuel oxidation and respiration that impact negatively upon skeletal muscle energy homeostasis [2, 5–7]. Indeed, mitochondrial dysfunction and/or a reduction in mitochondrial content, as characterised by a fall in oxidative capacity, has been reported in skeletal muscle and adipose tissue from obese and diabetic subjects [8, 9]. Notably, the sustained oversupply of metabolic fuel (glucose and fatty acids) to skeletal muscle, as seen during Type II diabetes and obesity, impairs the ability of mitochondria to shift between use of lipid during fasting and use of carbohydrate in the post-prandial state. This metabolic inflexibility imposes a major substrate burden on the oxidative machinery of muscle and the continued oversupply of carbon fuel eventually surpasses the respiratory drive and cellular demand for ATP synthesis [10]. As a result, FAs undergo incomplete oxidation and greater partitioning into lipotoxic derivatives [e.g., diacylglycerol (DAG) and ceramides] which have been strongly implicated in the pathogenesis of insulin resistance [11, 12]. Whilst some have suggested that sufficient mitochondrial capacity remains under such circumstances [13], others submit that without energy demand such capacity is irrelevant and that metabolic dysfunction associated with the “effective” mitochondrial insufficiency may exacerbate effects of lipotoxicity [14–18].

Mitochondrial fuel overload also increases production of reactive oxygen species (ROS) [19, 20] that may serve to trigger or prime mechanisms that not only induce, but also sustain the loss in mitochondrial function. For example, it is has been suggested that increased ROS generation may suppress expression of PGC1α (a major regulator of mitochondrial biogenesis) and that of genes encoding components of the respiratory chain, but may also act as a stimulus activating proinflammatory NFkB signalling that intersects with processes influencing mitochondrial function [21, 22]. In support of this latter idea, signalling initiated by toll-like receptor-4 (TLR-4) and the tumor necrosis factor-α (TNF-α) receptor results in activation of the NFkB pathway, which has been linked to reduced mitochondrial respiration and suppressed activation of transcriptional regulators that promote mitochondrial biogenesis and the shift towards a muscle oxidative phenotype [23, 24]. We, and others, have also previously demonstrated that obesity in rodents and chronic oversupply of metabolic fuel to skeletal muscle cells in vitro is associated with an increase in proinflammatory NFkB signalling and insulin resistance [17, 25, 26]. We hypothesise that sustained oversupply of metabolic fuel will promote activation of proinflammatory NFkB signalling in muscle cells and that this contributes significantly to disturbances in mitochondrial biology that impact negatively upon myocellular insulin sensitivity. The studies reported herein have tested this proposition.

## Materials and methods

### Chemicals and reagents

Culture media α-MEM (α-minimum essential medium), DMEM (Dulbecco’s Modified Eagle’s medium), Medium 199, foetal bovine serum, horse serum, Mitosox, prolong diamond antifade mountant, and mitotracker dyes were all purchased from Thermo Fisher Scientific (UK). α-MEM media-lacking glucose was purchased from PAN Biotech, UK, Mitoquinone (Mito Q) was obtained from Cambridge biosciences, UK). BI605906 was a generous gift from Professor Sir Philip Cohen (MRC Protein Phosphorylation Unit, University of Dundee), but also purchased from Tocris (Bristol, UK), MitoSpy™ Green FM was from BioLegends, UK and MitoPYI, Mitotempo, hepatocyte growth factor, dexamethasone, basic FGF, gelatine, vitamin B12, retinoic acid, VAS2870, palmitate, oligomycin, FCCP (carbonyl cyanide *p*-trifluoromethoxyphenylhydrazone), rotenone, antimycin-A, hygromycin B, apo-transferrin human, SYBR^®^ Green JumpStart Taq Ready Mix, and Polyberen were all purchased Sigma-Aldrich, UK).

### Rat and human skeletal muscle cell culture, transfection and fatty acid treatment

L6 muscle cells were cultured to myotubes as described previously [27] in α-minimal essential media (αMEM) containing 2% (v/v) foetal bovine serum (FBS) and 1% (v/v) antibiotic/antimycotic solution (100 units/ml penicillin, 100 μg/ml streptomycin, and 250 ng/ml amphotericin B) at 37 °C with 5% CO_2_. In some experiments, L6 myotubes were infected with adenovirus harbouring a mutated IκBαS32A/S36A construct. This virus was kindly provided by Dr Harry Heimberg (Vrije Universiteit Brussel, Belgium) and was initially propagated in HEK293 cells and stored at – 80 °C. The viral titre was determined by standard plaque assay in HEK293 cells. Confluent mononucleated L6 myoblasts were infected with the adenovirus at 5.5 pfu/cell for IκBαS32A/S36A in serum free α-MEM for 2 h at 37 °C. Cells were subsequently maintained in fresh α-MEM containing 2% FBS at 37 °C and allowed to differentiate into myotubes prior to experimental use. For some studies, we also used human (LHCN-M2) myotubes. These were cultured in DMEM/M199 medium (4:1) supplemented with penicillin streptomycin (100 μg/ml), FBS 15% (v/v) HEPES (20 mM), Zinc sulphate (30 ng/ml), vitamin B12 (1.4 μg/ml), dexamethasone (55 ng/ml), hepatocyte growth factor, recombinant human (2.5 ng/ml), and basic FGF (10 ng/ml) on plastic ware that had been coated overnight with gelatin (0.1% (*w*/*v*) at room temperature. Confluent myotubes were differentiated by change of culture media to DMEM/medium 199 containing zinc sulphate, HEPES, vitamin B12, insulin (10 μg/ml), and apotransferrin (100 μg/ml) and refreshing this every 48 h for 9–10 days until myotubes were 70–80% differentiated.

Prior to use in experiments, L6 myotubes were incubated in serum-containing media that either had or lacked d-glucose (5 mM) as indicated in the figures. For palmitate (PA) treatments, a 100 mM stock solution of the fatty acid was prepared in absolute ethanol as previously reported [28, 29]. This stock was subsequently diluted to a final concentration as indicated in the figure legends by addition to culture media containing 2% (*w*/*v*) fatty acid-free BSA and allowed to precomplex for 1 h at 37 °C before being applied onto myotubes for the periods indicated in the figure legends.

### Quantitative real-time PCR, mitochondrial DNA quantification, and analysis of citrate synthase activity

Myotubes were incubated with glucose, palmitate, and 2-dexoglucose (2DG) or with inhibitors and/or fluorescent dyes as indicated in the figure legends and prepared for RNA extraction, qPCR analysis, and immunoblotting as described previously [17, 25, 30, 31]. Briefly, total RNA was extracted from L6 myotubes using the TRizol extraction protocol (Thermo Fisher Scientific, UK). RNA samples were used to prepare cDNA using a qScript cDNA synthesis kit as per manufacturer’s instructions and cDNA quantified using the real-time PCR Syber Green based method to establish mRNA abundance. Analysis of mitochondrial DNA (mtDNA) and citrate synthase activity were used as a proxy for mitochondrial mass. For mtDNA quantification, total DNA was extracted from L6 myotubes using a Qiagen DNaesy kit. The mtDNA was quantified by qPCR using primers directed against the mitochondrial ND4 gene and the nuclear-encoded COX4 gene and using the Syber Green method. Data were expressed as a ratio of the ∆∆*C*_t_ ND4 to the ∆∆*C*_t_ of COX4. The forward and reverse primer sequences for the different gene targets are detailed in Table 1. Citrate synthase (CS) activity was measured using a kit purchased from Sigma-Aldrich/UK (MAK193). Myotubes were treated as indicated in the figure legend and whole cell extracts prepared at the end of the appropriate treatments. 20 μg protein from the cell extract was used for each enzymatic analysis (with measurements being conducted in triplicate for each experimental determination at room temperature). Enzyme activity was measured spectrophotometrically (using an absorbance wavelength of 412 nm) using a µQUNT BIOTEK plate reader from LabTech UK with readings taken every 3 min over a 60 min assay period. CS activity was calculated as per manufacturer’s instructions.

**Table 1:** Primer sequences

GENE	Forward primer	Reverse primer
SDHA	GCCACTCACTCTTACACACC	GCACTCCCCATTTTCCATC
UCP3	GTCAAGCAGTTCTACACCCC	TTTCCTCTCGCCTCCAGTTC
ANT1	TCATCTACAGAGCTGCCTAC	TCATCATCCTACGACGGAC
IL6	AGCCACTGCCTTCCCTACTT	GCCATTGCACAACTCTTTTCTC
ND4	GAGGCAACCAAACAGAACGC	ATCATGTTGAGGGTAGGGGGT
COX4	AATGTTGGCTACCAGGGCAC	GGGTAGTCACGCCGATCAAC
β-ACTIN	TGGAGAAGATTTGGCACCACAC	CAGAGGCATACAGGGACAACAC
PGC1α	TGAACTACGGGATGGCAAC	AAGAGCAAGAAGGCGACAC

### Subcellular fractionation

A mitochondrial-enriched membrane fraction was isolated from L6 myotubes using a mitochondria isolation kit (#89874, Thermo Fisher Scientific) as per manufacturer’s instructions. The methodological protocol involves homogenisation of myotubes that have been harvested from 10 cm tissue culture plates having undergone prior experimental treatments as indicated in the appropriate figure legends in lysis buffer [10 mM HEPES, PH 7.5, 10 mM KCl, 0.1 mM EDTA, 0.1 mM DTT, 0.5% (v/v) Nonidet-P40 and 0.5 mM PMSF and protease inhibitor cocktail]. The homogenised cell material was subject to two differential centrifugation steps and within the final centrifugation step the resulting mitochondrial pellet was washed twice prior to being solubilised in RIPA buffer. The supernatant from the final spin (cytosolic fraction) and the solubilised mitochondrial membrane pellet were stored at – 20 ºC until required.

For isolation of nuclear membranes, L6 myotubes were grown on 10 cm dishes as described above and, after treatments, washed three times in PBS before being harvested and spun down in a microfuge (100 g for 5 min). The cell pellet was resuspended in lysis buffer and held on ice for 20 min with intermittent mixing prior to being centrifuged at 10,000 *g* for 5 min. The resulting supernatant represents a cytosolic fraction. The pelleted nuclei were washed three times in lysis buffer before being resuspended in nuclear extraction buffer (20 mM HEPES PH 7.5, 400 mM NaCl, 1 mM EDTA, 1 mM DTT, 1 mM PMSF with protease inhibitor cocktail) and re-spun at 10,000*g* for 15 min at 4 °C. The resulting nuclear pellet was resuspended in fresh extraction buffer and stored at – 20 °C until required.

### SDS-PAGE and immunoblotting

Cell lysates, cytosolic, nuclear, or mitochondrial-enriched fractions (20 μg protein) from L6 myotubes and human LHCN-M2 myotubes were subjected to SDS/PAGE on 10% resolving gels and transferred onto nitrocellulose membranes (Millipore, Harts, UK), as described previously [27]. Membranes were probed with the following primary antibodies for immunoblot analysis: actin (#A5060) and tubulin (#T6074) were obtained from Sigma: ANT-1 (#ab180715) and PGC1α (#ab54481) were from Abcam; IkBα (#SC-371), SDHA (#SC98253), and GAPDH (#SC32233) were purchased from Santa Cruz; p65 (#8242), Akt (#9272), p-AktSer473 (#9271S), TOM20 (# 42406S), HA (#2367S), COX4 (#4580S), and GPX1 (# 3286S) and SOD2 (#D9V9C) were all purchased from Cell Signalling Technology; DLP1/Drp1 (#611112) and OPA1 (#612607) were from BD Biosciences; and UCP3 (#GTX112699) from Genetex. Primary antibody detection was performed using appropriate horse-radish peroxidase (HRP) conjugated secondary mouse (#7076S) or rabbit (#7074S) antibodies were purchased from Cell Signalling Technology and visualised using enhanced chemiluminescence (Pierce-Perbio Biotech, Tattenhall, UK) on Kodak X-OMAT film (Eastman-Kodak, Rochester, UK). The immunoreactive protein bands were quantified using ImageJ software.

### Glucose uptake

L6 myotubes were incubated with glucose, palmitate and BI605906 for times and at concentrations indicated in the figure legends prior to assaying uptake of 10 μM 2-deoxy-d-[^3^H]-glucose as described previously [27]. Non-specific binding was determined by quantifying cell-associated radioactivity in the presence of 10 μM cytochalasin B. Cells were washed and subsequently lysed in 50 mM NaOH and radioactivity quantified by scintillation counting. Protein concentration in cell lysates was determined using the Bradford reagent [32].

### ROS quantification

For analysis of superoxide, L6 myotubes were subject to experimental treatments as indicated in the figure legends prior to being treated with 5 μM Mitosox at 37°C in a 5% CO_2_ incubator for 30 min. Mitosox is a fluorogenic dye that is specifically targeted to mitochondria in live cells, and whose oxidation by superoxide produces red fluorescence that was quantified using a Clario Star plate reader with absorption/emission maxima: 510/585 nm. In some experiments, L6 myotubes were also treated with Mitotempo (a mitochondrial targeted anti-oxidant) prior to analysis of superoxide.

For determination of hydrogen peroxide (H_2_O_2_) under live cell conditions, L6 myotubes were incubated with 5 μM MitoPYI (a mitochondrial targeted H_2_O_2_ probe) and 1 μM deep red cell tracker at 37 °C in a 5% CO_2_ incubator for 45 min. Myotubes were subsequently imaged using a Zeiss confocal microscope with excitation/emission maxima for MitoPYI set to 488/530 nm and that for the cell tracker at 633/647 nm. Captured images were analysed to quantify the fluorescent signal generated by MitoPYI from at least 8–10 different visual fields (40–50 myotubes) per condition per experiment using ImageJ software.

### Analysis of cellular respiration and mitochondrial energetics

For analysis of cellular respiration and mitochondrial energetics in L6 and LHCN-M2 myotubes, we used a Seahorse XF24 analyser. L6 myotubes were cultured on Seahorse culture plates in serum-containing media supplemented with 5 mM d-glucose and/or palmitate at concentrations indicated in the figure legends for 16 h. In some experiments, the culture media were also supplemented with 5 mM 2-deoxyglucose (2-DG), BI605906 (IKKβ inhibitor) or 2 mM carnitine as indicated prior to analysis of basal respiration, ATP-linked respiration, H+ (proton) leak, maximal respiratory capacity and non-mitochondrial respiration using modulators of cellular respiration (i.e., oligomycin, FCCP (carbonyl cyanide *p*-trifluoromethoxyphenylhydrazone), rotenone, and antimycin as previously described [33]. The various mitochondrial parameters were normalised to protein content/well within the Seahorse plate. For Seahorse XF analyser studies, data points per experimental condition were collected from a minimum of three replicates with each experiment being conducted at least three times.

### Mitochondria morphology and live cell mitochondrial imaging

For analyses of mitochondrial morphology, we stained L6 myotubes with Mitospy Green FM (BioLegend, UK); a green-fluorescent stain that localizes to mitochondria. L6 myotubes were grown on 15 mm^2^ glass coverslips and following the experimental treatments specified in the figure legends were washed with fresh media and subsequently incubated in medium containing 300 nM Mitospy for 30 min at 37°C in a 95% O_2_/5% CO_2_ environment. After this incubation period, myotubes were washed with PBS prior to being fixed with 2% (*w*/*v*) paraformaldehyde and mounted in prolonged diamond antifade before being visualised using a Zeiss confocal microscope. Live cell imaging was also used in some of our studies. For these, L6 or LHCN-M2 myotubes were grown and differentiated in eight well chamber slide plates (Ibidia, UK) and having been treated (as indicated in the figure legends) were washed with fresh phenol red-free media prior to incubation with Mitospy. Mitochondrial morphology was then visualised in real time using Zeiss confocal microscope 37 °C in a 5% CO_2_ chamber with excitation/emission set at 480 nm and 520 nm, respectively. For real-time recording of mitochondrial length, we used the ZEISS ZEN microscope software or Image J. Within each experimental condition, at least 50 myotubes were randomly selected from between 10 and 12 visual fields. Mitochondrial morphology within myotubes was categorised as either spheroid/fragmented in which mitochondria were equal to or less than 1 μm in length or tubular/elongated (including being part of a network), where mitochondrial length was greater than 1 μm. The number of mitochondria in each category within the fields being visualised was then determined and expressed as a percentage.

### Analysis of mitophagy

Mitophagy was quantified using the mitophagy QC approach [34], which involves stable expression of a tandem mCherry-GFP tag attached to the outer mitochondrial membrane localization signal of Fis1 (residues 101–152) [34]. The retrovirus harbouring this construct was introduced into L6 myotubes using the approach detailed previously [35]. The L6-GFP–mCherry cells were grown and differentiated on 15 mm^2^ cover slips and subjected to the treatments detailed in the figure legends prior to being washed and fixed with 3.7% cell culture grade paraformaldehyde and mounted in prolonged diamond antifade. Cells were visualised using Zeiss 710 confocal microscope. Myotubes expressing the mCherry-GFP construct fluoresce red and green (yellow when confocal images are merged). However, upon increased mitophagy, mitochondria are delivered to lysosomes, where the low pH quenches the GFP signal but not mCherry. Consequently, some of the mitochondria form punctate structures and fluoresce red only and the degree of mitophagy calculated by quantitating their increase using the volocity software.

### Statistical analysis

Statistical analysis was performed using the GraphPad Prism version 7 software using one-way analysis of variance (ANOVA) and Tukey post hoc test for multiple comparisons. Values were considered significant at *P* < 0.05.

## Results

### Effects of palmitate oversupply on proinflammatory signalling, ROS generation and mitochondrial function in myotubes

**Fig. 1:**
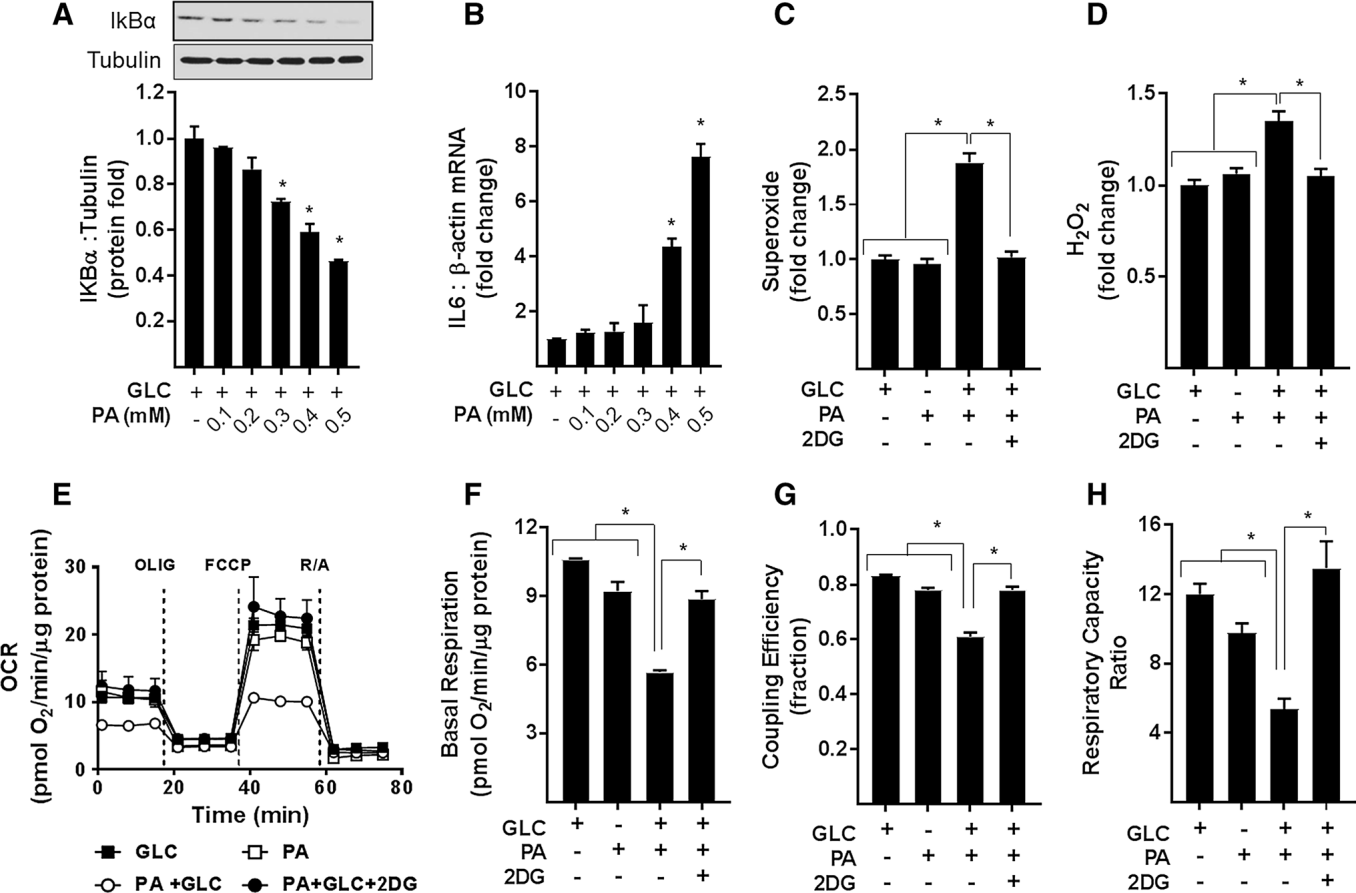
Cellular fuel overloading induces NFkB inflammatory signalling, ROS (superoxide and H_2_O_2_) production and mitochondrial dysfunction. L6 myotubes were incubated with glucose (GLC, 5 mM) in the absence and presence of palmitate (PA) at doses indicated for 16 h prior to analysis of (**a**) cellular IkBα abundance by immunoblotting and **b** IL6 and β-actin mRNA abundance by qPCR. For analysis of superoxide (**c**) and H_2_O_2_ (**d**) L6 myotubes were treated as in (**a**) with PA (0.4 mM) for 16 h in absence or presence GLC (5 mM) and/or 2-deoxyglucose (2DG, 5 mM) as indicated followed by quantification of ROS using 5 μM of either Mitosox or MitoPYI as detailed in methods. For analysis of real-time cellular respiration in L6 myotubes we used a Seahorse XF24 analyser. L6 myotubes were incubated for 16 h with either GLC (5 mM) or PA (0.4 mM) alone, PA (0.4 mM)/GLC (5 mM) together or with a mixture of PA (0.4 mM)/GLC (5 mM)/2DG (5 mM). Oligomycin (1 μM), FCCP (1 μM) and a rotenone (1 μM)/antimycin-A (2 μM) mix were added at times indicated by dotted lines on the Seahorse trace. The trace shown in (**e**) is a representative readout of oxygen consumption rate (OCR) from a single experiment with measurements (mean ± SD) of triplicate values. Analysis of absolute basal mitochondrial OCR normalised to protein/well (**f**), coupling efficiency of oxidative phosphorylation (**g**, determined as the oligomycin sensitivity fraction of the basal respiratory rate) and (**h**) the respiratory capacity ratio (calculated as a factor of the FCCP-stimulated respiration/oligomycin resistance). The data shown in (**f**–**h**) is the combined analyses of three separate experiments. All data are presented as mean ± SEM. Asterisks indicate a significant change (*P* < 0.05) to the GLC alone condition or between the indicated bars

In an attempt to establish the relationship between changes in proinflammatory NFkB signalling, ROS generation, and mitochondrial biology, we initially investigated the effects of modulating palmitate (PA) provision on these parameters. L6 myotubes were incubated in α-MEM containing a physiological (5 mM) d-glucose (GLC) concentration in the absence and the presence of increasing concentrations of palmitate (PA; 0.1–0.5 mM) for 16 h. At the end of this period, the abundance of IkBα and expression of IL-6 mRNA were monitored as readouts of NFkB signalling. Figure 1a, b shows that myotubes exposed to increasing PA concentrations exhibit a dose-dependent reduction in cellular IkBα abundance that was associated with a concomitant increase in IL-6 gene expression. Since the threshold concentration at which PA induced a significant change in IkBα and IL-6 gene expression was 0.4 mM, all subsequent experiments involving nutrient overloading of myotubes were conducted in the presence of 5 mM GLC and 0.4 mM PA unless otherwise indicated. It is important to stress that combined provision of GLC and PA at these concentrations did not invoke any notable death or loss of terminally differentiated myotubes on culture plates.

A characteristic feature associated with mitochondrial fuel overload is increased cellular ROS [e.g., superoxide (O_2_^–.^) and hydrogen peroxide (H_2_O_2_)], whose generation can promote oxidative damage and metabolic dysfunction. Whilst incubation of myotubes with 5 mM GLC or 0.4 mM PA alone had no significant effect on ROS production in myotubes, the combined presence of both nutrients induced a significant increase in O_2_^–.^ and H_2_O_2_ (Fig. 1c, d). The notion that this increase in ROS is driven by nutrient overload is strengthened by our demonstration that increases in O_2_^–.^ and H_2_O_2_ are regulated in a PA and GLC concentration-dependent manner (Supplementary Fig. S1 and S2) and that increases in ROS can be restrained in myotubes exposed to 2-deoxyglucose (2DG); a glycolytic inhibitor that severely restricts GLC use as a metabolic fuel (Fig. 1c, d; Supplementary Fig. S1 and S2). Furthermore, we contest that the increase in ROS, we see in response to GLC/PA overloading is principally mitochondrial generated given that treating myotubes with either Mitotempo or MitoQ, two mitochondrial targeted anti-oxidants, which, respectively, scavenge O_2_^–.^ and quench H_2_O_2_, effectively suppress increases in both ROS initiated by fuel oversupply (Supplementary Fig. S1D and S2D).

**Fig. 2:**
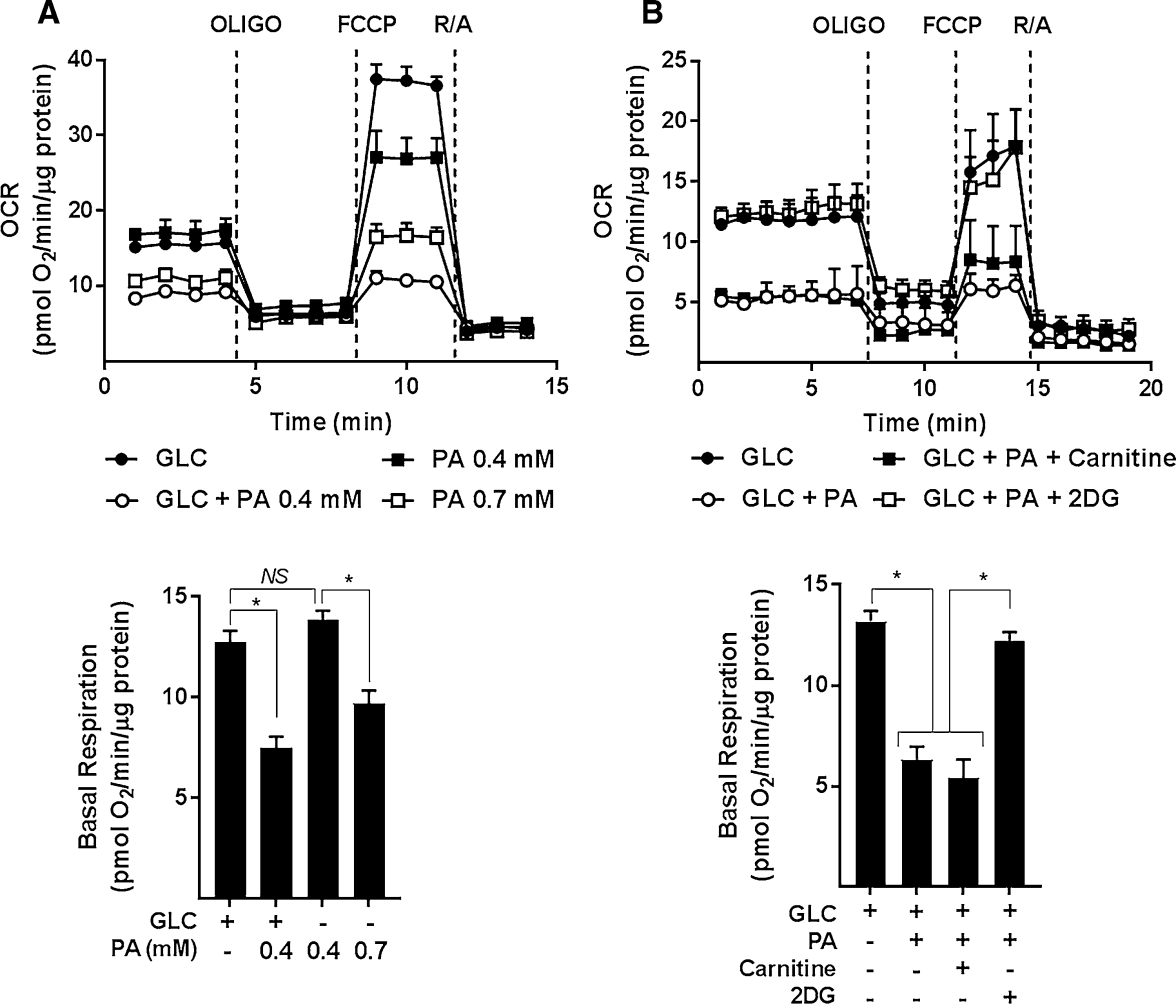
Effect of glucose/palmitate overloading and carnitine supplementation on mitochondrial function in L6 myotubes. L6 myotubes were incubated with glucose (GLC, 5 mM), palmitate (PA, 0.4 mM or 0.7 mM) or with GLC (5 mM) and PA (0.4 mM) together for 16 h as shown in (**a**) or in some experiments (**b**) when treated with GLC and PA together such treatments were done in the presence of either carnitine (2 mM) or 2-deoxyglucose (2DG, 5 mM) prior to analysis of real-time cellular respiration using a Seahorse XF24 analyser. All data are presented as mean ± SEM. Asterisks indicate a significant change (*P* < 0.05) between the indicated bars. *NS* signifies no significant change

For analysis of real-time mitochondrial respiration in myotubes, we utilised a Seahorse extracellular flux analyser that measures oxygen consumption rates (OCR) before and after addition of compounds that target Complexes I and III of the respiratory chain, the ATP synthase or which function to uncouple mitochondrial oxidative phosphorylation (OXPHOS) to allow analysis of numerous mitochondrial parameters. Figure 1e–h shows that compared to myotubes incubated with either 5 mM GLC or 0.4 mM PA alone, those exposed to both carbon fuels simultaneously had a lower OCR and also exhibited a significant reduction in basal respiration, OXPHOS coupling, and respiratory capacity. Strikingly, these respiratory changes are averted in myotubes incubated in media containing GLC and PA, but to which 2DG was also added to inhibit glucose use as a metabolic substrate. To further demonstrate that this amelioration most likely reflects a lowering of mitochondrial substrate load rather than a reduction in substrate competition, we assessed the impact of increasing the PA concentration in the absence of any GLC. Figure 2a shows that whilst the OCR was comparable when myotubes were incubated with either 5 mM GLC or 0.4 mM PA alone and co-provision of GLC and PA at these concentrations induced a significant decline in basal respiration, this reduction could also be recapitulated in myotubes incubated with PA alone, but when presented at a higher concentration (0.7 mM) to increase mitochondrial substrate load. It is also important to stress that incubation of myotubes with PA was conducted in serum-containing media in which carnitine was present at physiological concentrations. Consequently, we believe it unlikely that carnitine would be limiting for mitochondrial uptake and oxidation of PA at the concentrations used in our studies. In line with this view, exogenous supplementation of carnitine (2 mM) to culture media did not enhance or ameliorate the reduction in mitochondrial respiratory capacity that we see in PA/GLC treated myotubes (Fig. 2b).

### Effects of mitochondrial fuel overload and glycolytic inhibition on mitochondrial morphology

**Fig. 3:**
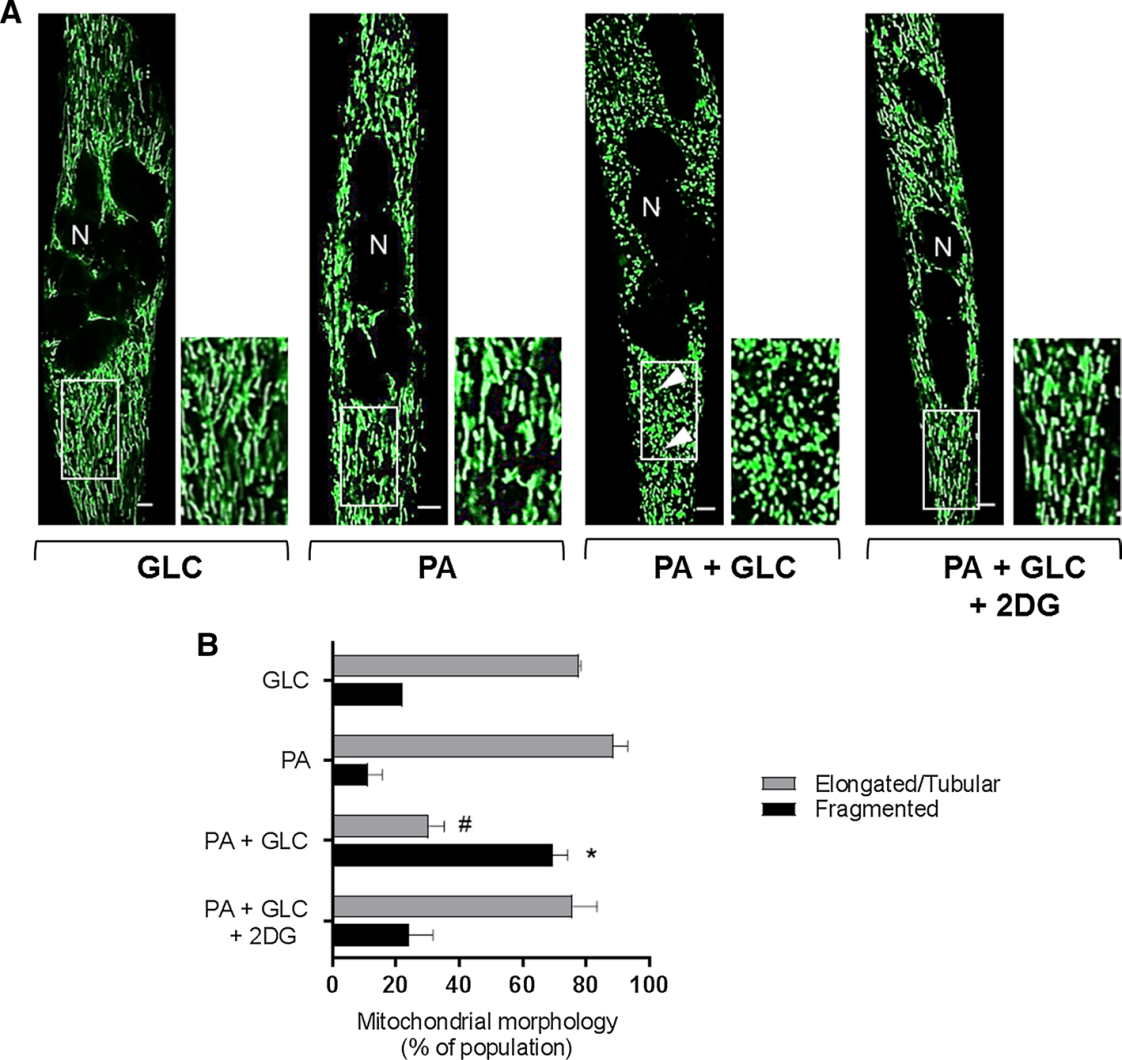
Effect of glucose/palmitate overloading on mitochondrial morphology in L6 myotubes. L6 myotubes were incubated for 16 h with either GLC (5 mM) or PA (0.4 mM) alone, PA (0.4 mM)/GLC (5 mM) together or with a mixture of PA (0.4 mM)/GLC (5 mM)/2DG (5 mM) prior to staining with Mitotracker green (Mitospy) and confocal microscopy. **a** Confocal images depict mitochondrial morphology in L6 myotubes (the scale bar represents 5 μm). Nuclei (N) are labelled and white boxed areas are magnified to show differences in morphology. The arrow heads depict fragmented mitochondria. **b** Mitochondrial length was quantified using the imaging software and presented as elongated/tubular if greater than 1 μm and fragmented if < 1 μm in length. Data are presented as mean + SEM from a minimum of three separate experiments. Asterisks indicate a significant change (*P* < 0.05) between the black filled bars, whereas the hash (#) indicates a significant difference (*P* < 0.05) between the grey filled bars

The data presented in Fig. 1e–h imply that chronic oversupply of metabolic fuel (GLC + PA) to myotubes impairs mitochondrial respiration and that this can be mitigated when use of GLC as a metabolic fuel was restricted using 2DG as a glycolytic inhibitor. To test whether the reduced respiratory function assayed under fuel-overload conditions was associated with changes in mitochondrial morphology, we subsequently performed live cell imaging to visualise mitochondria using Mitotracker Green. The dye is excluded from nuclei, but accumulates within mitochondria and consequently helps depict the syncytial nature of differentiated L6 myotubes used in our study. Strikingly, in myotubes that had been incubated with GLC or PA alone, the green-fluorescent mitochondrial dye highlights that ~ 80% of the mitochondrial population is part of an organised elongated/tubular network, which becomes structurally fragmented and spheroid in nature when myotubes are subjected to a sustained period of fuel overloading with GLC and PA (Fig. 3a, b). In line with the finding that glycolytic inhibition with 2DG helps preserve mitochondrial respiratory function (Fig. 1e–h), the morphological change in the mitochondrial network caused by substrate overloading was mitigated by 2DG (Fig. 3).

### Is proinflammatory NFkB signalling a driver of ROS generation in response to nutrient overloading?

**Fig. 4:**
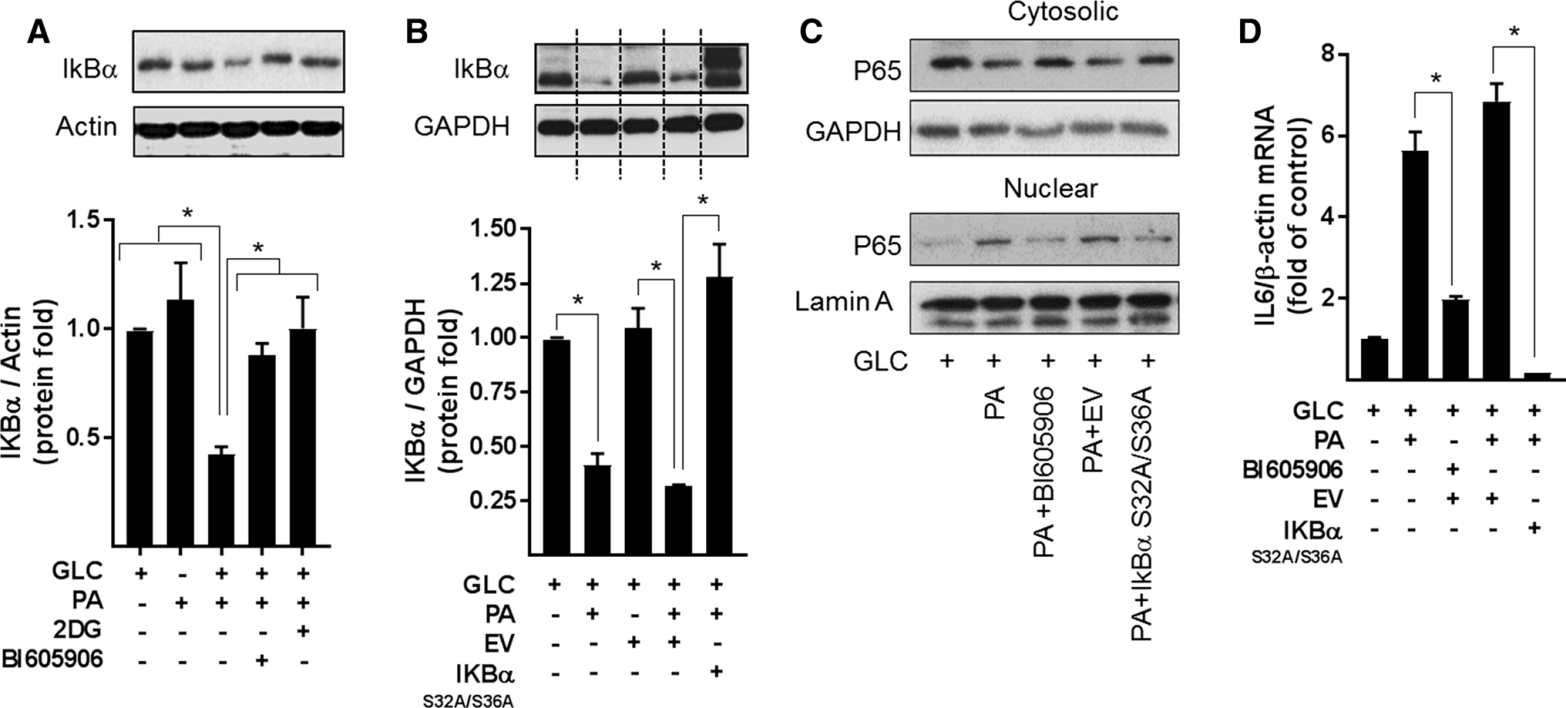
Effects of suppressing NFkB on inflammatory signalling induced by nutrient overload in L6 myotubes. **a** L6 myotubes were incubated for 16 h with either GLC (5 mM) or PA (0.4 mM) alone, PA (0.4 mM)/GLC (5 mM) together or with a mixture of PA (0.4 mM)/GLC (5 mM)/2DG (5 mM) in the absence or presence of BI605906 (10 μM), an IKKβ inhibitor prior to analysis of cellular IkBα abundance by immunoblotting. **b** L6 myoblasts were infected with an adenoviral vector expressing HA-tagged non-phosphorylatable IkBα (S32A/S36A) or one expressing an empty vector (EV). Cells were allowed to differentiate prior to treatment of cells with GLC, PA and 2DG and analysis of IkBα abundance as described in (**a**). L6 myotubes were treated with GLC (5 mM), PA (0.4 mM), BI605906 (10 μM) and adenoviral vectors as indicated prior to (**c**) subcellular fractionation and analyses of cytosolic and nuclear NFkB p65 abundance by immunoblotting or **d** analysis of IL-6 gene expression. All data are presented as mean ± SEM from a minimum of three separate experiments. Asterisks indicate a significant change (*P* < 0.05) between the indicated bars

To explore whether the increase in NFkB signalling seen in myotubes subjected to nutrient overload is a contributing factor to mitochondrial ROS production and respiratory dysfunction, we applied two distinct but complimentary strategies. The first utilised BI605906, a potent pharmacological inhibitor that shows high selectivity for IKKβ (IC_50_ = 380 nM) when tested against over 100 kinases in a panel screen [36], whereas the second involved adenoviral mediated expression of a mutant form of IκBα (S32A/S36A) that functions as a super-repressor of NFκB activity [25]. Mutation of Ser32 and Ser36 to alanine renders IκBα resistant to IKK phosphorylation, thereby protecting it from proteasomal degradation and retaining its capacity to hold NFκB in an inhibited state. To test the effectiveness of each strategy, we initially assayed the ability of BI605906 and the IκBα^S32A/S36A^ mutant to suppress NFkB activation in response to nutrient overload in L6 myotubes. The data in Fig. 4a, b show that the reduction in IkBα instigated by a 16 h period of nutrient (GLC + PA) overloading in myotubes was halted by BI605906 or by cellular expression of the IkBα mutant. Moreover, consistent with this observation, both BI605906 and IκBα^S32A/S36A^ expression attenuated nuclear localisation of the p65 subunit of NFkB and reduced transcription of the IL-6 gene (an NFkB target gene) that is otherwise seen in GLC+PA-overloaded myotubes (Fig. 4c, d).

**Fig. 5:**
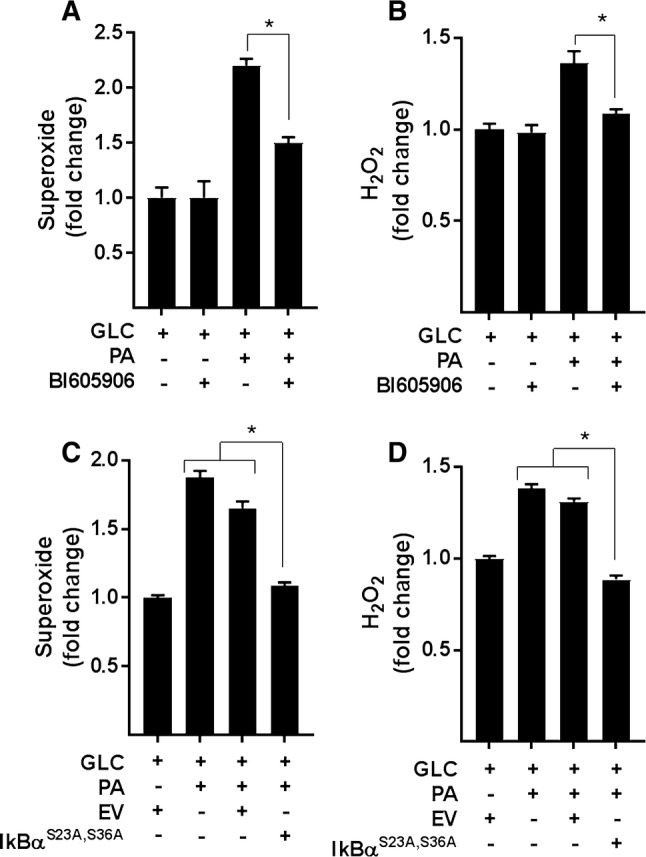
Effects of NFkB antagonism on ROS production. **a**, **b** L6 myotubes were incubated for 16 h with either GLC (5 mM) or PA (0.4 mM) alone, PA (0.4 mM)/GLC (5 mM) together in the absence or presence of BI605906 (10 μM) or subject to these treatments having been infected with an adenoviral vector expressing HA-tagged non-phosphorylatable IkBα (S32A/S36A) or one expressing an empty vector (EV) **c**, **d** prior to analysis of superoxide and hydrogen peroxide. All data are presented as mean ± SEM from four separate experiments. Asterisks indicate a significant change (*P* < 0.05) between the indicated bars

Analysis of ROS production in L6 myotubes revealed that in the absence of PA provision, BI605906 *per se* has no detectable effect upon cellular O_2_^–.^ or H_2_O_2_, but significantly reduces the increase in both species when myotubes were subjected to chronic nutrient oversupply (Fig. 5a, b). Similarly, repressing NFkB activation by expression of IκBα^S32A/S36A^ blocked the increase in ROS triggered by nutrient overload (Fig. 5c, d). It is noteworthy that this nutrient-induced increase in ROS is accompanied by an attendant increase in the expression of anti-oxidant enzymes [superoxide dismutase 2 (SOD2), catalase and glutathione peroxidase (GPX1)], which most likely forms part of a cellular defence mechanism designed to help limit oxidative damage/stress under these circumstances (Supplementary Fig. S2E). Intriguingly, however, whilst the elevated ROS generation induced by cellular over-nutrition was restrained by BI605906 (Fig. 5a, b), the inhibitor did not to suppress the increased expression of SOD2, catalase or GPX1 (Supplementary Fig. S2E), suggesting that expression of these enzymes was likely to be regulated by mechanisms that are distinct to those involved in promoting generation of O_2_^–^ and H_2_O_2_.

### Is increased NFkB signalling a causal factor for mitochondrial dysfunction in myotubes during nutrient overload?

**Fig. 6:**
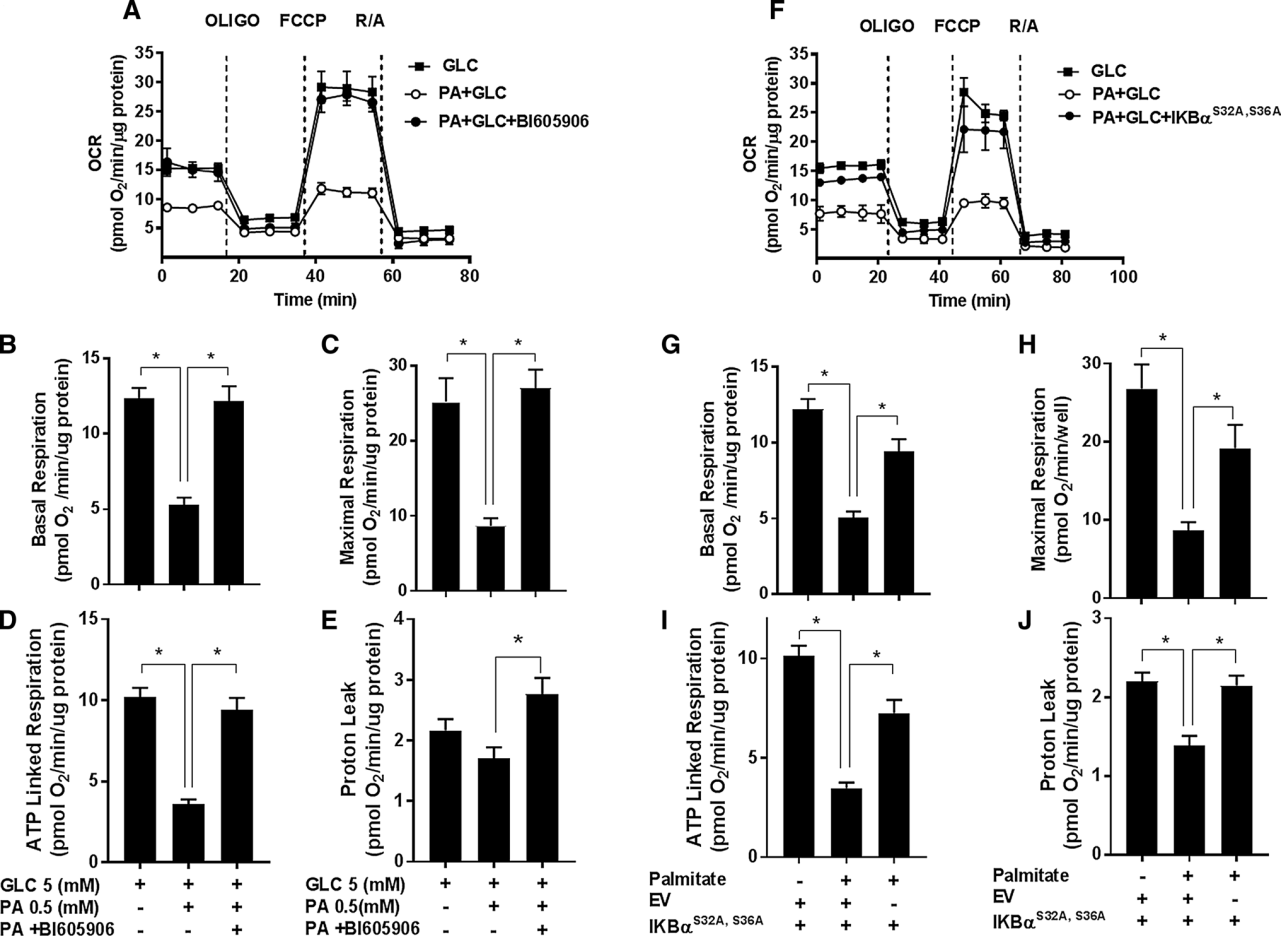
Effect of suppressing NFkB activation in response to cellular fuel overloading on mitochondrial respiration. **a–e** L6 myotubes were incubated for 16 h with either GLC (5 mM) or PA (0.4 mM) alone, PA (0.4 mM)/GLC (5 mM) together in the absence or presence of BI605906 (10 μM) or **f**–**j** subject to these treatments having been infected with an adenoviral vector expressing HA-tagged non-phosphorylatable IkBα (S32A/S36A) or one expressing an empty vector (EV) prior to analysis of real-time cellular respiration in L6 myotubes using a Seahorse XF24 analyser. Oligomycin (1 μM), FCCP (1 μM) and a rotenone (1 μM)/antimycin-A (2 μM) mix were added at times indicated by dotted lines. **a**, **f** show representative readouts of oxygen consumption rate (OCR) from a single experiment with measurements (mean ± SD) of triplicate values. **b**, **g** Depict basal mitochondrial oxygen consumption rate (OCR), **c**, **h** maximal respiration (OCR after FCCP stimulation), **d**, **i** ATP-linked respiration (oligomycin-sensitive OCR) and **e**–**j** proton leak (oligomycin resistance rate). Data are presented as mean ± SEM from five independent experiments. Asterisks indicate a significant change (*P* < 0.05) between the indicated bars

Impaired mitochondrial bioenergetics can result in the excessive generation of ROS during the process of OXPHOS. Since inhibition of NFkB signalling suppresses the increase in cellular ROS associated with nutrient overload, we subsequently tested whether this might be linked to improved mitochondrial function. The data in Fig. 6 show the effects of inhibiting NFkB signalling in L6 myotubes (using BI605906 and by cellular expression of the IκBα^S32A/S36A^ super-repressor) upon mitochondrial function. In line with the findings presented in Fig. 1e–h, sustained (16 h) exposure of myotubes to nutrient excess (GLC and PA) induced a significant decline in the basal and maximal respiratory rate, which was associated with a reduction in ATP-linked respiration and a modest decline in mitochondrial proton leak (Fig. 6a–j). Strikingly, these nutrient-induced disturbances in mitochondrial respiration were ameliorated if activation of the IKKβ-NFkB signalling axis by nutrient excess was repressed by BI605906 in a dose-dependent manner (Fig. 6a–e and Supplementary Fig. S3) or expression of the IκBα^S32A/S36A^ mutant (Fig. 6f–j). Notably, the improved respiratory drive that we see under these circumstances is associated with a significant increase in mitochondrial proton leak (Fig. 6e, j) potentially signifying increased mitochondrial uncoupling that would also help offset ROS generation.

### Effects of repressing NFkB signalling on mitochondrial morphology and expression of proteins important for mitochondrial function

**Fig. 7:**
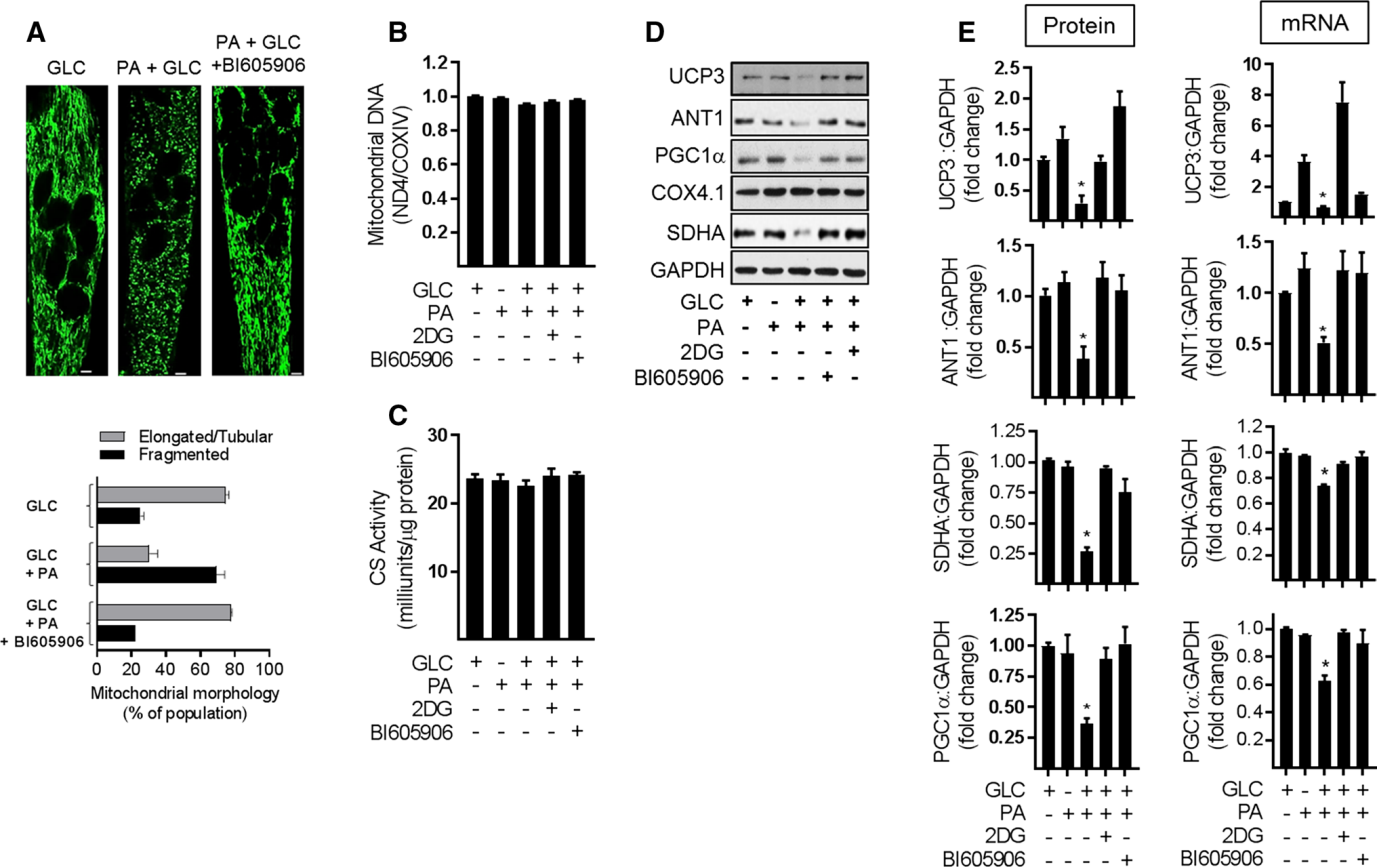
Effect of suppressing NFkB activation in response to cellular fuel overloading on mitochondrial morphology, mitochondrial proteins and gene expression. L6 myotubes were incubated with GLC (5 mM), PA (0.4 mM), 2DG (5 mM) and BI605906 (10 μM) for 16 h in the combinations indicated in the various experimental data panels prior to **a** analysis and quantification of mitochondrial morphology using Mitotracker green (Mitospy) by confocal microscopy (the scale bar represents 5 μm), **b** mitochondrial DNA copy number by qPCR, **c** citrate synthase (CS) activity and (**d**, **e**), analysis of mitochondria protein and mRNA abundance (UCP3, ANT1, PGC1α, SDHA, and COX4.1) which was normalised to GAPDH. All graphical bar data are presented as mean ± SEM from four separate experiments. Asterisks indicate a significant change (*P* < 0.05) to the GLC alone condition

To assess whether the improved respiratory capacity that was seen upon inhibiting NFkB signalling in nutrient-overloaded myotubes was associated with changes in mitochondrial morphology or mitochondrial mass we subsequently performed live cell imaging of mitochondria stained with Mitotracker Green and also assessed cellular mitochondrial DNA content. Figure 7a shows that repressing NFkB signalling with BI605906 helps attenuate the fragmentation of the elongated/tubular mitochondrial network that is otherwise seen when myotubes are chronically exposed to nutrient excess. qPCR analysis was used to determine mitochondrial DNA copy number by quantifying the relative abundance of the mitochondrial encoded NADH dehydrogenase 4 (ND4) gene to that of COX4 (a nuclear-encoded gene). Figure 7b, c shows that irrespective of whether myotubes were subject to nutrient overload or not, or whether they were treated with 2DG or BI605906, we observed no significant differences in mitochondrial DNA or citrate synthase activity (a representative nuclear-encoded mitochondrial enzyme) in response to the various experimental manipulations. Both these measures are considered a proxy of mitochondrial mass.

Whilst mitochondrial mass in L6 myotubes was unaltered following a 16 h period of nutrient overload, the reduced respiratory rate seen under these circumstances was associated with a significant reduction in the abundance of proteins with key roles in mitochondrial bioenergetics. These include uncoupling protein 3 (UCP3), mitochondrial ADP–ATP translocase (ANT1) and succinate dehydrogenase (SDHA) as well as PGC1α, which has a major role in regulating mitochondrial biogenesis and function (Fig. 7d). Our analysis reveals that the decline in these proteins may have been driven by a decrease in gene expression based on the reduced mRNA abundance that, respectively, encode the four proteins (Fig. 7e). Whilst we saw no change in the protein abundance of Cox 4.1 (a subunit of Cytochrome C-oxidase that functions within the mitochondrial respiratory chain) in myotubes exposed to both GLC and 0.4 mM PA, we have shown previously that its cellular abundance declines dramatically when the presence of PA in GLC-containing media is raised above 0.4 mM [17, 31]. This latter finding may potentially signify that the expression and stability of Cox4.1 may have a slightly higher tolerance for nutrient stress than some of the other mitochondrial proteins that we have investigated. Significantly, however, the loss seen in UCP3, ANT1, SDHA, and PGC1α protein in fuel-overloaded myotubes was ameliorated by not only pharmacological repression of the IKKβ-NFkB signalling axis with BI605906, but also by restraining GLC metabolism using 2DG (Fig. 7d, e).

It is plausible that the increased respiratory drive seen in myotubes that have been subject to fuel overloading, but in which activation of the NFkB pathway has been blunted (Fig. 6) is a benefit derived from suppressing mitochondrial ROS production (Fig. 5). However, our analysis indicates that whilst exposing myotubes to two distinct mitochondrial targeted anti-oxidants (Mitotempo and Mito Q) limits fragmentation of the mitochondrial network induced by nutrient oversupply, neither compound could rescue the loss in UCP3, ANT1, or PGC1α expression or prevent the decline in mitochondrial respiratory capacity (Supplementary Fig. S4). However, we did see some recovery in the expression of SDHA. We are mindful that ROS production can also occur at extra-mitochondrial sites such as via NADPH oxidase (NOXII) in the cytosol, but targeting NOXII with a cell permeable inhibitor, VAS2870, also proved ineffective in countering the decline in respiratory capacity caused by nutrient excess (Supplementary Fig. S4).

### Effects of mitochondrial substrate overload on mitophagy

**Fig. 8:**
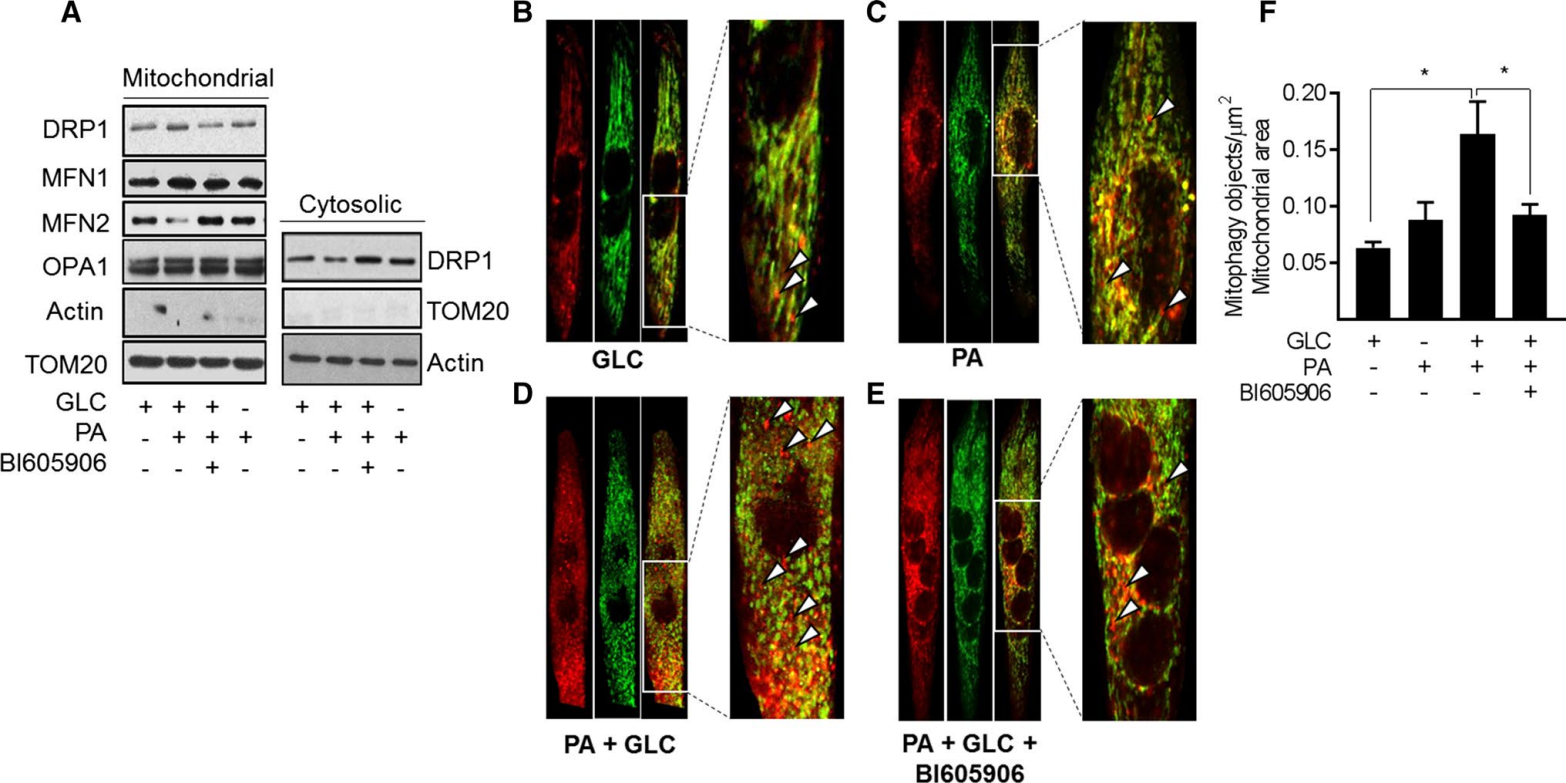
Effects of cellular fuel overloading on mitophagy and upon proteins linked to mitochondrial dynamics. Wild type L6 myotubes or those stably expressing a retroviral vector encoding a GFP–mCherry–Fis1 mitophagy reporter were incubated with GLC (5 mM), PA (0.4 mM) and BI605906 (10 μM) for 16 h as indicated prior to: **a** subcellular fractionation and isolation of a cytosolic and mitochondrial-enriched membrane fraction for immunoblotting with antibodies to proteins shown. **b**–**e** Fixing and confocal imaging to visualise and **f** quantifying (using the Volocity software) mitophagy in L6 myotubes. Data (mean ± SEM) in (**f**) are from five separate experiments. Asterisks indicate a significant change (*P* < 0.05) between the indicated bars. Boxed sections in panels **b**, **c**, **d** and **e** have been expanded to highlight mitophagic particles, some of which are depicted by the white arrow heads

Since chronic oversupply of metabolic fuel promotes mitochondrial dysfunction, we postulated that under such circumstances, there might be an increase in mitophagy to help clear damaged/dysfunctional mitochondria. To test this hypothesis, we initially monitored expression of proteins implicated in the control of mitochondrial dynamics in a mitochondrial-enriched membrane fraction isolated from myotubes that had been subject to fuel overloading in the absence and presence of BI605906. Figure 8a shows that sustained exposure of myotubes to 5 mM GLC and 0.4 mM PA induced a very modest increase in the mitochondrial abundance of Drp1, a GTPase that facilitates mitochondrial fission. This increase in mitochondrial-associated Drp1 may have resulted from its recruitment from a cytoplasmic pool, which showed a corresponding decline in its abundance. By contrast, we noted a loss in mitochondrial mitofusin 2 (MFN2), a GTPase resident on the outer mitochondrial membrane involved in mitochondrial clustering and fusion (Fig. 8a). The relative changes in mitochondrial Drp1 and MFN2 is consistent with a shift in mitochondrial dynamics that favours increased fission and would be in line with our morphological analysis (Fig. 7a). In contrast, the observed changes in mitochondrial-associated Drp1 and MFN2 abundance were repressed in fuel-loaded myotubes that had been treated with BI605906 and would fit with the reduced mitochondrial fragmentation that we see (Fig. 7a). No notable differences were observed for mitofusin 1 (MFN1) or optic atrophy-1 protein (OPA-1) in the mitochondrial and cytosolic fractions that we examined (Fig. 8a).

To assess whether increased fragmentation/fission in response to nutrient excess also increases mitophagy, we utilised myotubes stably expressing a tandem mCherry-GFP tag attached to the outer mitochondrial membrane localization signal of Fis1 (residues 101–152) [34]. Figure 8b, c shows representative field images that highlight (red) the presence of mitophagic objects in myotubes incubated with GLC or PA alone, which most likely reflects basal mitophagy (see arrow heads within the magnified regions from the box inserts). Whilst this approach does not quantitatively assess mitophagic flux, it indicates that the combined provision of GLC and PA induces a significant increase in mitophagic particles (Fig. 8d, f) whose appearance is restrained upon cotreatment of myotubes with BI605906 (Fig. 8e, f).

### Effects of suppressing NFkB signalling in substrate-loaded myotubes on insulin sensitivity

**Fig. 9:**
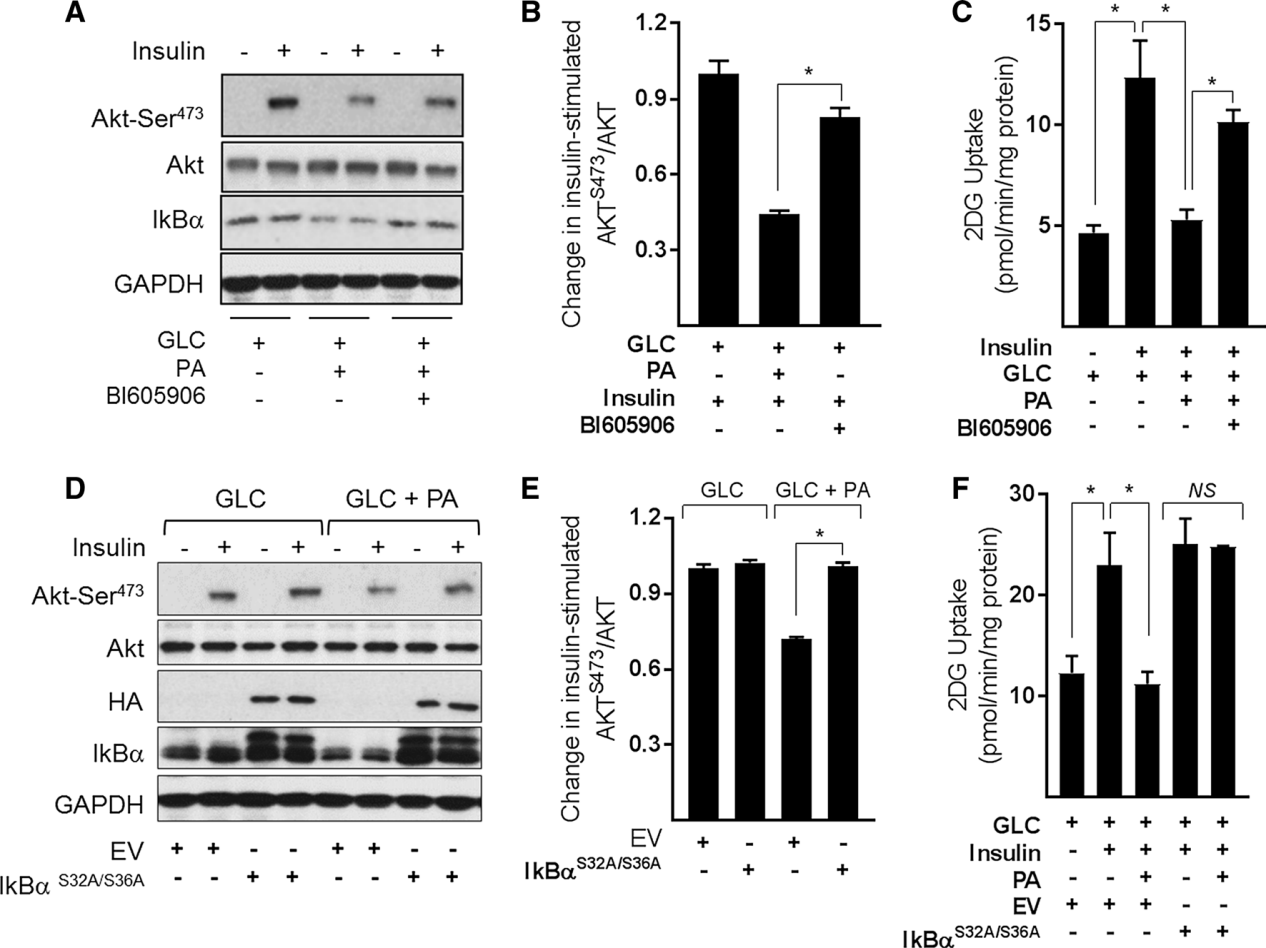
Effects of suppressing NFkB signalling in response to cellular fuel overloading on insulin-stimulated PKB phosphorylation and glucose uptake in L6 myotubes **a**–**c** L6 myotubes were incubated for 16 h with GLC (5 mM) or PA (0.4 mM) alone or with PA (0.4 mM)/GLC (5 mM) together in the absence or presence of BI605906 (10 μM). Alternatively, **d**–**f** muscle cells were subject to incubation with GLC and PA as indicated having been infected with an adenoviral vector expressing HA-tagged non-phosphorylatable IkBα (S32A/S36A) or one expressing an empty vector (EV) prior to acute stimulation with insulin (20 nM) for 15 min (Akt signalling experiments) or insulin (100 nM) for 20 min for glucose uptake studies. Following insulin stimulation, cells were either lysed for immunoblotting using antibodies to proteins indicated (**a**, **d**) or used for assay of glucose uptake. The data are mean ± SEM. from 5 separate experiments. Asterisks indicate a significant change (*P* < 0.05) between the indicated bars

A number of previous studies have shown that sustained exposure of muscle cells to nutrient (glucose and fatty acid) excess impairs insulin action and have linked this to disturbances in mitochondrial function. To assess whether preserving the respiratory capacity and integrity of the mitochondrial network in nutrient overloaded myotubes by inhibition of NFkB signalling improves insulin action, we assessed insulin-stimulated Akt phosphorylation and glucose uptake as readouts. Figure 9a shows that insulin induces a robust increase in Akt^Ser473^ phosphorylation/activation that is blunted significantly (by ~ 58%) in myotubes that have been subject to GLC/PA oversupply (Fig. 9b). Associated with this loss in Akt-directed insulin, signalling was a substantial loss in insulin-stimulated glucose uptake (Fig. 9c). However, cotreatment of fuel-loaded myotubes with BI605906 or expression of the IκBα^S32A/S36A^ super-repressor (Fig. 9d) not only mitigates the reduction in insulin-stimulated Akt phosphorylation (Fig. 9a, b, d, e), but also partially rescues the loss in hormone-stimulated glucose uptake (Fig. 9c, f).

## Discussion

We have previously shown that heightened NFkB signalling associated with sustained nutrient oversupply, as occurs during obesity, plays an important role in lipid-induced insulin resistance and metabolic dysfunction in skeletal muscle both in vitro and in vivo [17]. Precisely how an increase in NFkB signalling mechanistically links to changes in muscle insulin action is poorly understood, but we postulate that disturbances in mitochondrial homeostasis, driven by an increase in proinflammatory signalling, may potentially represent an important component within this link. This proposition is based on the view that whilst mitochondria play a crucial role in balancing energy supply with demand under normal circumstances, they become functionally compromised when supply of metabolic fuel chronically exceeds cellular energy demand. Our results indicate that the sustained oversupply of both GLC and PA imposes a major substrate burden on mitochondria in L6 myotubes and that this promotes (1) a reduction in mitochondrial respiratory capacity, (2) reduced expression of key mitochondrial proteins, (3) increased generation of ROS and (4) increased mitochondrial fragmentation and mitophagy. It is also important to highlight that these observations are not just restricted to L6 myotubes, but can also be demonstrated in LHCN-M2 myotubes; a myogenic cell line obtained from skeletal muscle of a healthy male human subject (Supplementary Fig. S5). These nutrient-induced changes collectively signal a marked loss in mitochondrial integrity/function that impact negatively upon myocellular energy metabolism and, by extension, upon processes involved in fuel utilisation, such as glucose uptake and its regulation by insulin. Strikingly, our data indicate that pharmacological or genetic inhibition of the IKK–NFkB axis not only obviates detrimental changes in mitochondrial morphology and function, but also helps ameliorate disturbances in insulin signalling and insulin-dependent glucose uptake. Furthermore, allied to these observations, it is worth stressing that withholding either GLC or PA from the media or inhibiting GLC use as a metabolic fuel in the presence of PA not only averts activation of the NFkB pathway but antagonises mitochondrial fragmentation and the loss in respiratory function (Figs. 1b, 3a, 4a). These findings imply that whilst myotubes can efficiently metabolise GLC (5 mM) or PA (0.4 mM) when presented independently at the concentrations indicated, their combined carbon load appears to overwhelm the respiratory capacity of myotubes, especially if it is not matched by any increase in energy demand. Under these circumstances, the over provision of fatty acids leads to their incomplete oxidation and accumulation as short chain acyl-carnitines  and their greater partitioning into lipotoxic fatty acid derivatives (e.g., DAG and ceramide) that have been implicated in the development of muscle insulin resistance [6, 11, 37–39]. The production of ceramide and certain DAG species may, in turn, feedback to further suppress the activity of respiratory chain complexes [40, 41], which, associated with the reduced expression of succinate dehydrogenase (complex II) that is induced in response to chronic fuel excess, is likely to impair electron transfer within the respiratory chain. This proposition is supported by our finding that uncoupled respiration in the presence of FCCP was markedly lower in myotubes incubated with GLC/PA than those treated with either nutrient alone (Fig. 1e) It is, therefore, plausible that the observed perturbations in mitochondrial homeostasis seen in this study may, in part, be a consequence of cellular lipotoxicity initiated by the accumulation of molecules such as DAG and ceramide that would be associated with sustained oversupply of fatty acids.

Numerous studies have shown that saturated fatty acids, such as PA, have the capacity to induce IKK–NFkB signalling and can do so via a number of mechanisms including, for example, activation of toll-like receptors [42], MAP kinases (JNK and ERK) [25], excessive ROS generation [43] as well as by enhanced secretion of inflammatory cytokines (e.g., TNFα, IL-1β, and IL-6) that act via their respective cell surface receptors [44]. Whilst evidence exists associating these inflammatory cytokines with impaired mitochondrial function in some cell types [45, 46], studies linking classical NFkB signalling to mitochondrial dysfunction in muscle cells in response to fuel over loading are extremely sparse. However, one potential mechanism by which NFkB may modulate myocellular energy metabolism is via its well-established role as a regulator of gene expression [47]. Notable NFkB gene targets include those encoding mitochondrial proteins or transcription factors that regulate expression of key mitochondrial genes. For example, recent work in human glioblastoma cells has highlighted that activated NFkB can bind to two response elements within the gene promoter of the ADP/ATP translocase, ANT-1, resulting in its reduced expression and concomitant decline in mitochondrial ATP production [48]. NFkB activation also represses the activity of PGC1α [49], which functions as a critical regulator of not only mitochondrial biogenesis/respiration but expression of nuclear respiratory factors (NRFs) that, in turn, modulate expression of mitochondrial proteins such as UCP3 and SDHA [50–52]. An increase in mitochondrial biogenesis/content may serve as an initial response to nutrient excess, but such compensation typically fails in the face of sustained nutrient oversupply. This compensation process appears to be very short lived in our in vitro myotube cultures as whilst we observe an initial increase in the gene expression of PGC1α, SDHA, ANT-1, and UCP3 (during the first 4 h of nutrient oversupply), we find that this rapidly declines over the succeeding 20 h myotube incubation period with GLC and PA (Supplementary Fig. S7). This decline correlates with an associated increase in NFkB activation as judged by the loss in IkBα and increased expression of IL-6 that is observed from 4 h onwards in myotubes (Supplementary Fig. S7). The notion that these events are likely to be linked is supported by our finding that the targeted inhibition of the IKK–NFkB axis helps mitigate the loss of these mitochondrial proteins and the associated decline in respiratory function. Assessing whether the changes in PGC1α, SDHA, ANT-1 and UCP3 that we see in myotubes in response to fuel overloading are primarily a consequence of a reduction in gene expression or also involve post-transcriptional modulation of processes such as protein synthesis and degradation represent important investigative goals of future work.

Another critical regulator of mitochondrial metabolism and cellular responsiveness to insulin is ROS [53]. Mitochondria are major sources of ROS and generated as a consequence of electron “slippage” within the electron transport chain (ETC) to oxygen during OXPHOS [54]. ROS generated by the ETC are considered important for preserving the normal biological functionality of the respiratory chain. However, under certain circumstances, including mitochondrial substrate overload, sustained and elevated generation of ROS supersedes the capacity of anti-oxidant defence mechanisms leading to increased oxidative stress. Components of the respiratory chain are highly susceptible to oxidative damage and this not only reduces the fidelity and operational efficiency of the ETC, but also results in further augmentation in ROS generation that effectively establishes a viscous cycle driving greater mitochondrial dysfunction [55]. Our findings reveal that heightened ROS production is indeed a feature of mitochondrial substrate overload (Fig. 5) and that this occurs despite an attendant increase in the expression of SOD2, GPX1 and catalase (Supplementary Fig. S2) that most likely represent a cellular stress response designed to limit oxidative stress. Intriguingly, whilst exposing GLC/PA-overloaded myotubes to mitochondrial targeted anti-oxidants such as Mitotempo restrains superoxide accumulation (Supplementary Fig. S1) and confers protection against mitochondrial fragmentation (Supplementary Fig. S4), treatment of myotubes with either Mitotempo or MitoQ could not rescue the decline in mitochondrial respiratory capacity induced by fuel overloading. This observation is most likely explained by the fact that neither anti-oxidant was able to mitigate activation of the IKK–NFkB pathway that we believe initiates the loss of key mitochondrial proteins, such as SDHA, UCP3, and ANT-1 in fuel-overloaded myotubes (Supplementary Fig. S4). SDHA is a critical component within both the TCA cycle and ETC, and consequently, its loss will impact negatively upon both mitochondrial processes. Whilst the precise functional role of UCP3 remains uncertain, its expression in cultured myotubes and rodent skeletal muscle has been linked to modulation of fatty acid oxidation [56–58], mitochondrial integrity, and to management of ROS/oxidative stress [59–62]. Likewise, in addition to catalysing the exchange of cytoplasmic ADP for mitochondrial ATP, ANT-1 can mediate fatty acid-induced uncoupling via its ability to transfer fatty acid anions across the inner mitochondrial membrane [63]. Consequently, mild uncoupling mediated by both UCP3 and ANT-1 may be beneficial in protecting mitochondria against the reducing pressure created by transient increases in fuel supply and, by doing so, restraining the excessive generation/accumulation of ROS that may otherwise promote oxidative damage and impair skeletal muscle insulin sensitivity. The fact that suppressing NFkB activation in fuel-overloaded myotubes with BI605906 or 2DG antagonises loss of UCP3 and ANT-1 and that, under these circumstances, we not only see a measurable increase in mitochondrial proton leak, but observe improved insulin sensitivity in L6 myotubes (Fig. 9) and in muscle of obese Zucker rats [17] is fully congruent with this view. Furthermore, it is noteworthy that the notion that mitochondrial expression/activity of ANT-1 may influence insulin sensitivity of muscle cells is supported by studies in cultured C2C12 myotubes, in which partial silencing of ANT-1 results in reduced cellular sensitivity to fatty acid-induced uncoupling and a significant reduction in insulin-stimulated glucose uptake [63].

In skeletal muscle, reduced mitochondrial respiration has also been linked to aberrant control of mitochondrial dynamics [64–67]. Increased exposure of myotubes to PA and fatty acid derivatives such as ceramide promote a low mitochondrial fusion to fission ratio resulting in fragmented, discontinuous mitochondria that not only exhibit diminished capacity for respiration and ATP synthesis, but also is thought to be causally linked to impaired insulin sensitivity and metabolic function [66–68]. Consistent with these observations, GLC/PA overloading of L6 myotubes in our studies induced a profound morphological change in mitochondria from an elongated/tubular network to one that is highly fragmented, indicative of a dynamic shift towards greater fission. The increase in Drp1 (a profission protein) and reduction in MFN2 (a profusion protein) that we see within a mitochondrial-enriched membrane fraction is fully consistent with this view. Strikingly, pharmacological inhibition of the IKK–NFkB axis in GLC/PA-overloaded myotubes not only suppresses the relative changes in Drp1 and MFN2, but reduces fragmentation of the mitochondrial network, in which, significantly, we find was also associated with amelioration in cellular respiratory capacity and insulin action. Since Mitotempo is able to partially restore the tubular mitochondrial network, but not the loss in respiratory capacity in fuel-overloaded myotubes (Supplementary Fig. S4), we postulate that increased fission is likely to be secondary to the increased production of ROS that stems from mitochondrial dysfunction induced by activation of the NFkB pathway. It is also noteworthy that whilst our studies indicate that the activation of canonical NFkB signalling initiated in response to fuel overloading promotes mitochondrial fragmentation, this process can also be influenced by non-canonical NFkB signalling, which is independent of IKKβ and IKKγ (NEMO) but dependent on IKKα dimers. Although we saw no differences in OPA-1 expression in our mitochondrial-enriched membrane fractions from fuel-overloaded L6 myotubes, MEF cells lacking IKKα (but not IKKβ) exhibit reduced cellular expression of OPA-1 and display enhanced mitochondrial fission [69]. It is also noteworthy that the activation of the classical NFkB pathway in C2C12 myotubes can result in suppressed IKKα expression and that this has implications for expression of OXPHOS genes [70]. Regardless of the stimulus promoting a shift towards greater fission, whilst some of the fragmented mitochondria may retain their functional capacity, the structural integrity of others is likely to be compromised (as reflected by an overall reduction in cellular respiratory capacity) and, if not recoverable, will be targeted for clearance by mitophagy. Consistent with this view, we observed a significant increase in the number of mitophagic particles in myotubes subjected to GLC/PA loading, and in line with the protective effects, we suggest that the inhibition of canonical NFkB signalling confers upon mitochondrial integrity and function; this increase in mitophagy was blunted in myotubes that had been cotreated with BI606906. It is worth stating that the observed increase in mitophagy and the associated loss of key mitochondrial proteins (SDHA, ANT-1, and UCP3) that we see in fuel-overloadeded myotubes occur despite their being no significant change in mitochondrial DNA or citrate synthase activity. One potential explanation for this apparent paradox is that whilst our mitophagy reporter assay is highly sensitive and the loss in mitochondrial proteins may be accounted for by a reduction in the transcription and/or translation of their respective genes and products, analysis of mitochondrial DNA may also capture that present within mitochondria during the early stages of the mitophagic process. If so, this would lessen any potential differences in mitochondrial DNA between the treatments that we have performed.

In conclusion, our findings indicate that muscle cells subjected to sustained oversupply of metabolic fuel exhibit an increase in proinflammatory NFkB signalling that is mechanistically linked to diminished mitochondrial function as evidenced by our ability to antagonise the marked decline in respiratory capacity, expression of key mitochondrial maker proteins and the increased fission and mitophagy by pharmacological or genetic repression of the IKK–NFkB axis. Whilst very recent work has shown that sustained inhibition of NFkB can adversely affect muscle development and mitochondrial function during early life its repression has no detrimental effect in adult muscle [71]. Consequently, our observations would imply that therapeutic strategies that help restrain NFkB activation as seen during circumstances of energy excess, such as obesity and age-onset Type II diabetes, may help counter disturbances in mitochondrial homeostasis and impart beneficial effects upon skeletal muscle insulin sensitivity.

## Electronic supplementary material

Below is the link to the electronic supplementary material.

Supplementary material 1 (PDF 1080 kb)

## Abbreviations

GLC: Glucose
PA: Palmitate
FAs: Free fatty acids
DAG: Diacylglycerol
NFKB: Nuclear factor kappa-light-chain-enhancer of activated B cells
IL6: Interleukin-6
TNF: Tumor necrosis factor-α
IKKβ: Inhibitor of nuclear factor kappa-B kinase subunit beta
IKBα: Inhibitor of nuclear factor kappa-B
ROS: Reactive oxygen species
ANT-1: Adenine nucleotide translocase type 1
UCP3: Uncoupling protein 3
SDHA: Succinate Dehydrogenase
PGC1α: Peroxisome proliferator-activated receptor gamma coactivator 1-alpha
MitoQ: Mitoquinone
TLR-4: Toll-like receptor-4
2DG: 2-Deoxyglucose
FCCP: Carbonilcyanide *p*-trifluromethoxyphenylhydrazone
OPA1: Optic atrophy 1
MFN2: Mitofusin 2
DRP1: Dynamin-related protein 1
