# Supplementary material for: Proinflammatory NFkB signalling promotes mitochondrial dysfunction in skeletal muscle in response to cellular fuel overloading
Source: Cell Mol Life Sci. 2019 May 17;76(24):4887–904. doi: 10.1007/s00018-019-03148-8 (PMC6881256; doi:10.1007/s00018-019-03148-8)
Supplement: Supplementary file 1 — Supplementary material 1 (PDF 1080 kb) [file 18_2019_3148_MOESM1_ESM.pdf]

Nisr et al - Proinflammatory NFkB signalling promotes mitochondrial dysfunction in skeletal muscle in response to cellular fuel overloading

Supplementary Figures:

FIG S1: Analysis of superoxide content in L6 myotubes.

L6 myotubes were incubated in media containing glucose (GLC), palmitate (PA) or 2-deoxyglucose (2DG) at concentrations indicated (A-D) for 16 h prior to analysis of superoxide content as described in methods. In some experiments, a superoxide scavenger Mitotempo was added at doses indicated (D) 1h prior to treatment with PA and then present in combination with PA for the 16 h experimental duration. The data shown are combined analyses of 4-6 separate experiments. All data are presented as mean $\pm$ SEM. Asterisks indicate a significant change ($P < 0.05$) to the GLC alone condition or between the indicated bars.

FIG S2: Analysis of mitochondrial hydrogen peroxide (H_2O_2) in L6 myotubes

L6 myotubes were incubated in media containing glucose (GLC), palmitate (PA) or 2-deoxyglucose (2DG) at concentrations indicated (A-D) for 16 h prior to analysis of mitochondrial hydrogen peroxide (H_2O_2) content as described in methods. In some experiments, the effects of an antioxidant, MitoQ, on mitochondrial H_2O_2 production was assessed by adding MitoQ at doses indicated (D) 30 min prior to treatment with PA and then present in combination with PA for the 16 h experimental duration. Myotubes were incubated with GLC (5 mM) or in combination with PA at concentrations indicated or with BI605906 (10 μM) for 16 h to assess effect on the abundance of antioxidant enzymes (SOD2, Catalase and GPX1) and GAPDH by immunoblotting (E). The data shown (A-D) is the combined analyses of three separate experiments. All data are presented as mean $\pm$ SEM. Asterisks indicate a significant change ($P < 0.05$) to the GLC alone condition or between the indicated bars.

FIG S3: Effects of BI605906 on cellular IkBα abundance and cellular respiration in L6 myotubes.

L6 myotubes were incubated in media containing 5 mM glucose (GLC) or in combination with 0.4 mM PA for 16 h in the absence and presence of BI605906 at the concentrations indicated prior to cell lysis and analysis of (A) IkBα and GAPDH (used as a loading control) abundance by immunoblotting or (B-D) mitochondrial function using the seahorse XF24 technique.

FIG S4: Effects of antioxidant treatment on mitochondrial morphology and function in nutrient loaded L6 myotubes.

L6 myotubes were incubated for 16h in media containing glucose (GLC, 5 mM) alone or in combination with PA (0.4 mM) and antioxidants Mitotempo (20 μM), Mito-Q (250 nM) and the NADPH inhibitor VAS2870 (20 μM), as indicated. These antioxidants were

supplemented to the media at least 30 min before provision of PA and were then present for the entire duration of the fatty acid treatment. (A) Confocal imaging showing the effect of Mitotempo on mitochondrial morphology in fuel loaded myotubes. B) Effect of Mitotempo and MitoQ on the abundance of IkBα, PGC1α, ANT1, UCP3, SDHA and GAPDH (used as loading control). (C and D) Effects of Mitotempo, Mito-Q and NADPH inhibitor VAS2870, respectively, on mitochondrial function assessed using Seahorse XF24 technique.

FIG S5 Effects of glucose/palmitate oversupply on IkBα abundance, mitochondrial morphology and function in cultured LHCN human myotubes.

Differentiated (10 day) LHCN human myoutubes were incubated in media containing 5 mM glucose (GLC) or in combination with palmitate (PA) at concentrations indicated for 16h prior to analysis of (A) abundance of IkBα and GAPDH (used as loading control) by immunoblotting, (B) mitochondrial morphology by confocal microscope imaging and (C) mitochondrial function by the seahorse XF 24 technique. The data in (C) is from three separate experiments presented as mean $\pm$ SEM. The asterisk indicates a significant change ($P < 0.05$) to the GLC alone condition.

FIG S6: Time dependent changes in the IkBα abundance and gene expression in response glucose/palmitate overloading of L6 myotubes.

L6 myotubes were incubated in media containing glucose (GLC, 5 mM) and palmitate (PA, 0.4 mM) for the periods indicated to assess (A) abundance of IkBα and GAPDH (used as loading control) by immunoblotting and (B) IL6, PGC1α, SDHA, UCP3 and ANT1 gene expression by qPCR which was normalised to β-actin mRNA.

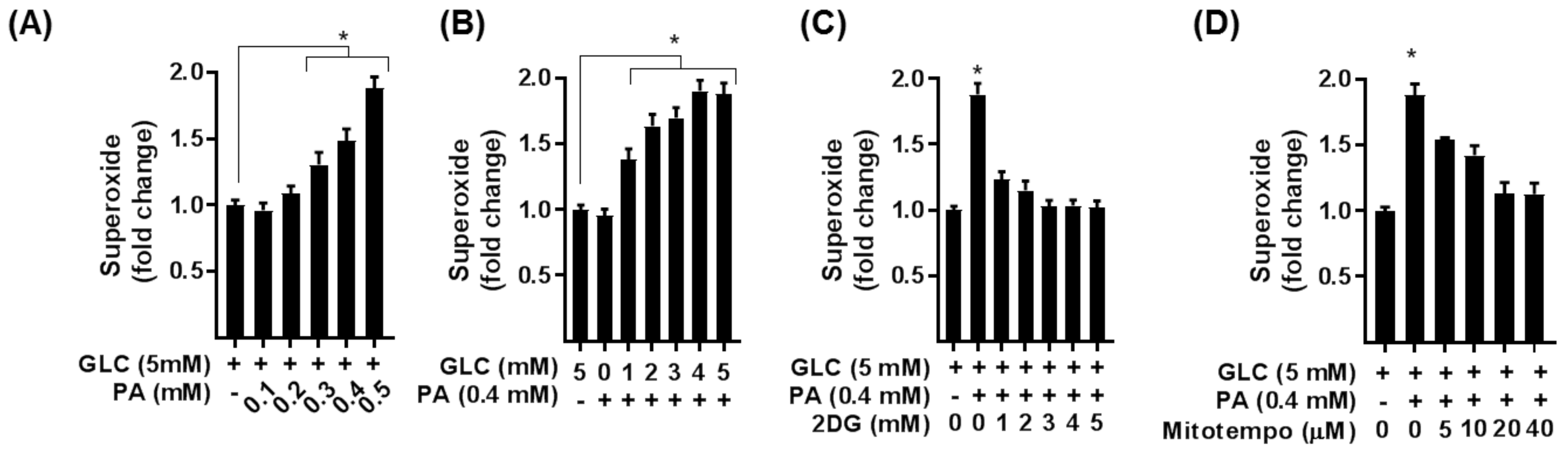
(A)
Superoxide (fold change)
2.0
1.5
1.0
0.5
*
GLC (5mM) + + + + + +
PA (mM) - 0.1 0.2 0.3 0.4 0.5
(B)
Superoxide (fold change)
*
GLC (mM) 5 0 1 2 3 4 5
PA (0.4 mM) - + + + + + +
(C)
Superoxide (fold change)
*
GLC (5 mM) + + + + + + +
PA (0.4 mM) - + + + + + +
2DG (mM) 0 0 1 2 3 4 5
(D)
Superoxide (fold change)
*
GLC (5 mM) + + + + + +
PA (0.4 mM) - + + + + +
Mitotempo (μM) 0 0 5 10 20 40

FIGURE S1

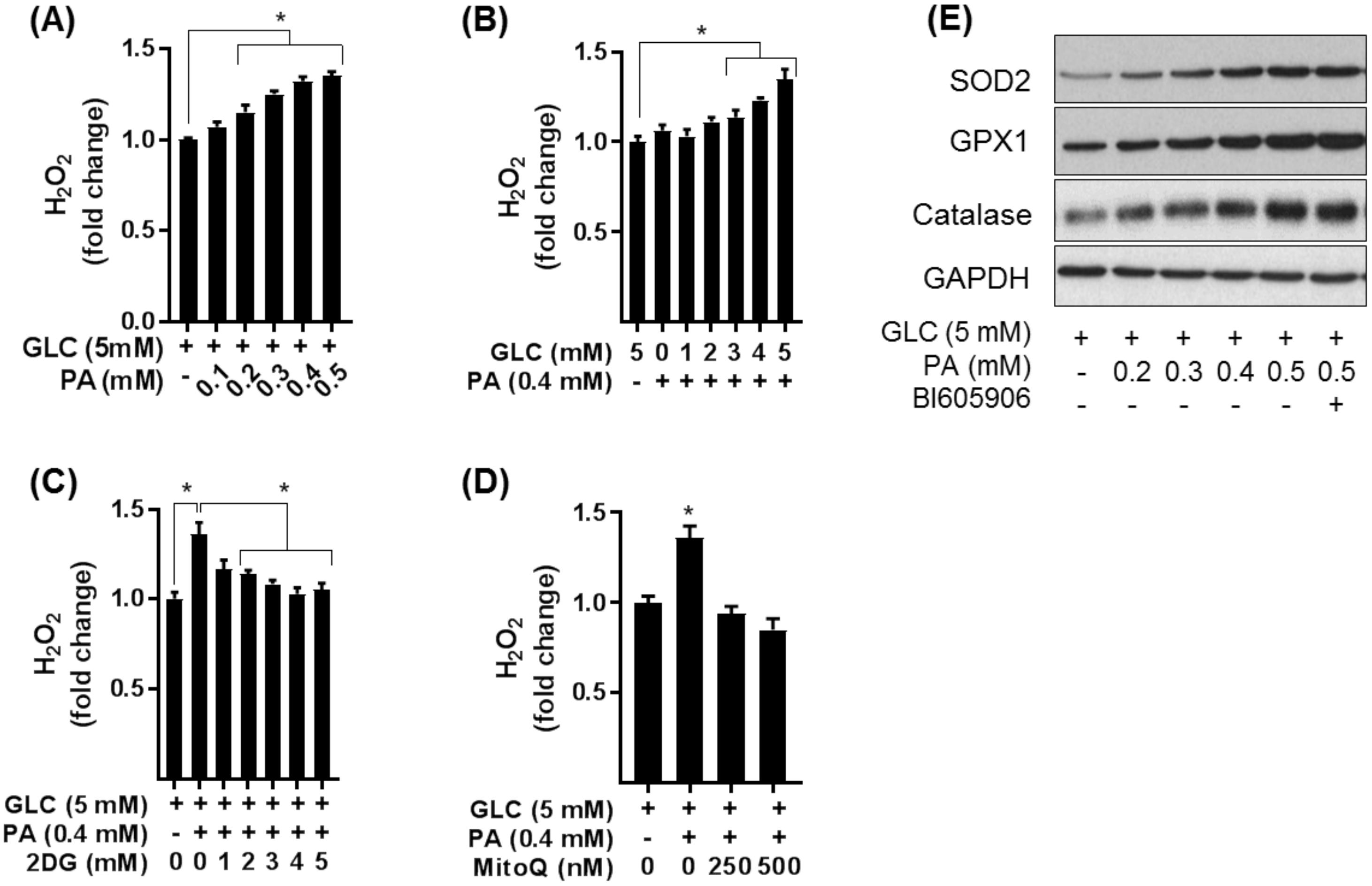
(A)
(B)
(C)
(D)
(E)
H2O2 (fold change)
1.5
1.0
0.5
0.0
*
GLC (5mM) + + + + + +
PA (mM) - 0.1 0.2 0.3 0.4 0.5
GLC (mM) 5 0 1 2 3 4 5
PA (0.4 mM) - + + + + + +
GLC (5 mM) + + + + + + +
PA (0.4 mM) - + + + + + +
2DG (mM) 0 0 1 2 3 4 5
GLC (5 mM) + + + +
PA (0.4 mM) - + + +
MitoQ (nM) 0 0 250 500
SOD2
GPX1
Catalase
GAPDH
GLC (5 mM) + + + + + +
PA (mM) - 0.2 0.3 0.4 0.5 0.5
BI605906 - - - - - +

FIGURE S2

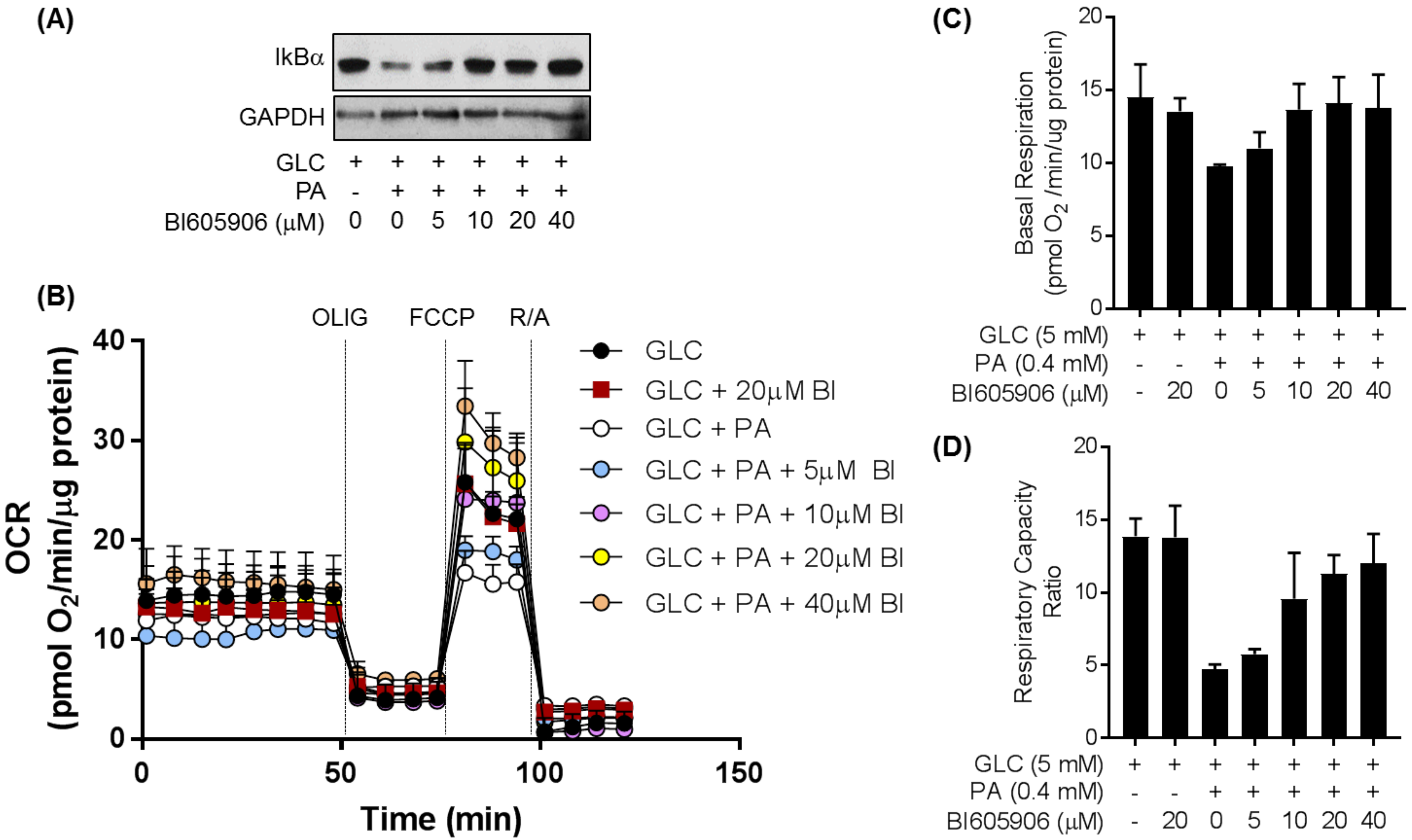

(A)
IkBα
GAPDH
GLC + + + + + +
PA - + + + + +
BI605906 (μM) 0 0 5 10 20 40
(B)
OLIG
FCCP
R/A
OCR (pmol O2/min/μg protein)
Time (min)
GLC
GLC + 20μM BI
GLC + PA
GLC + PA + 5μM BI
GLC + PA + 10μM BI
GLC + PA + 20μM BI
GLC + PA + 40μM BI
(C)
Basal Respiration (pmol O2 /min/ug protein)
GLC (5 mM) + + + + + + +
PA (0.4 mM) - - + + + + +
BI605906 (μM) - 20 0 5 10 20 40
(D)
Respiratory Capacity Ratio
GLC (5 mM) + + + + + + +
PA (0.4 mM) - - + + + + +
BI605906 (μM) - 20 0 5 10 20 40

FIGURE S3

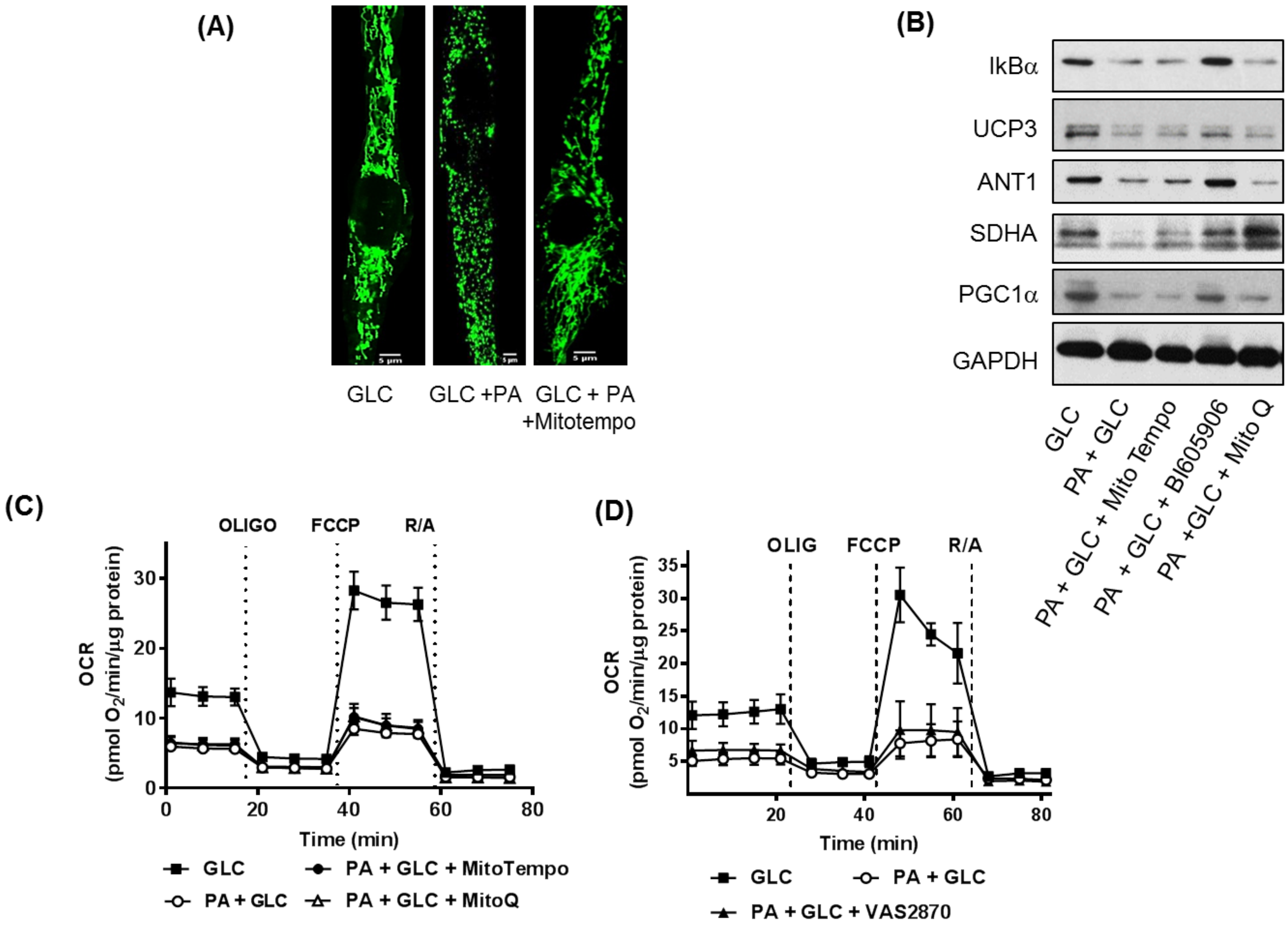

(A)
GLC
GLC +PA
GLC + PA +Mitotempo
(B)
IkBα
UCP3
ANT1
SDHA
PGC1α
GAPDH
GLC
PA + GLC
PA + GLC + Mito Tempo
PA + GLC + BI605906
PA +GLC + Mito Q
(C)
OLIGO
FCCP
R/A
OCR (pmol O2/min/μg protein)
Time (min)
GLC
PA + GLC
PA + GLC + MitoTempo
PA + GLC + MitoQ
(D)
OLIG
FCCP
R/A
OCR (pmol O2/min/μg protein)
Time (min)
GLC
PA + GLC
PA + GLC + VAS2870

FIGURE S4

Human Myotubes (LHCN)

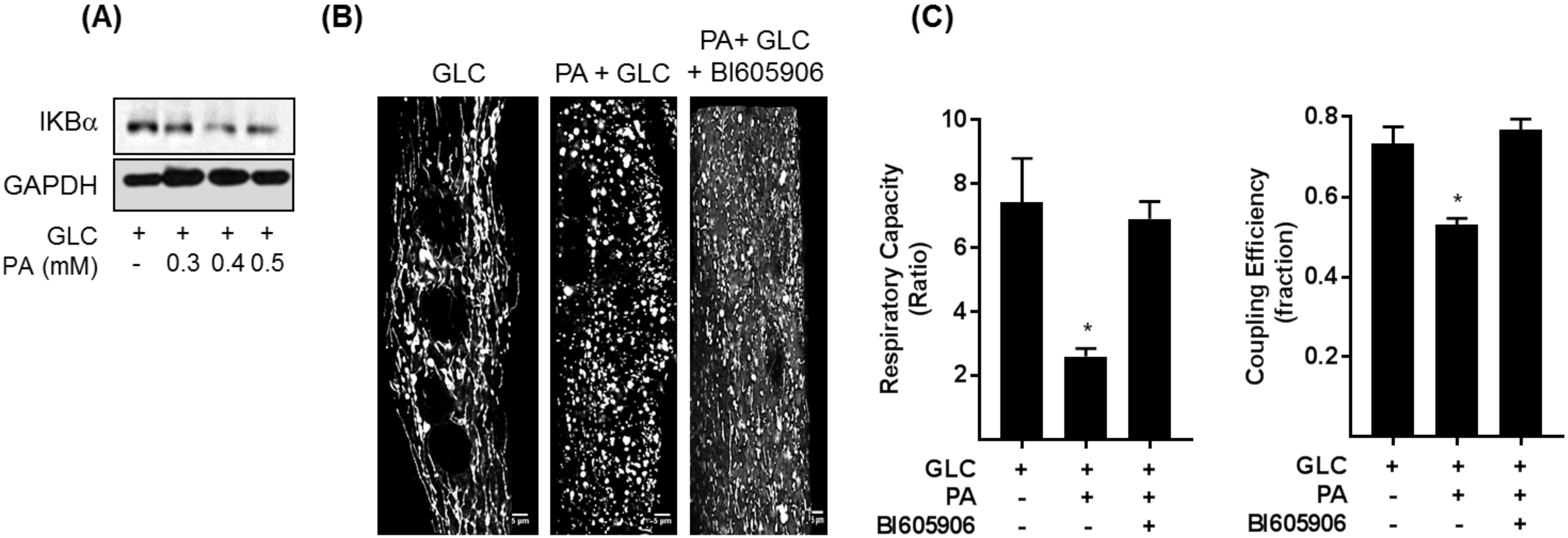

FIGURE S5

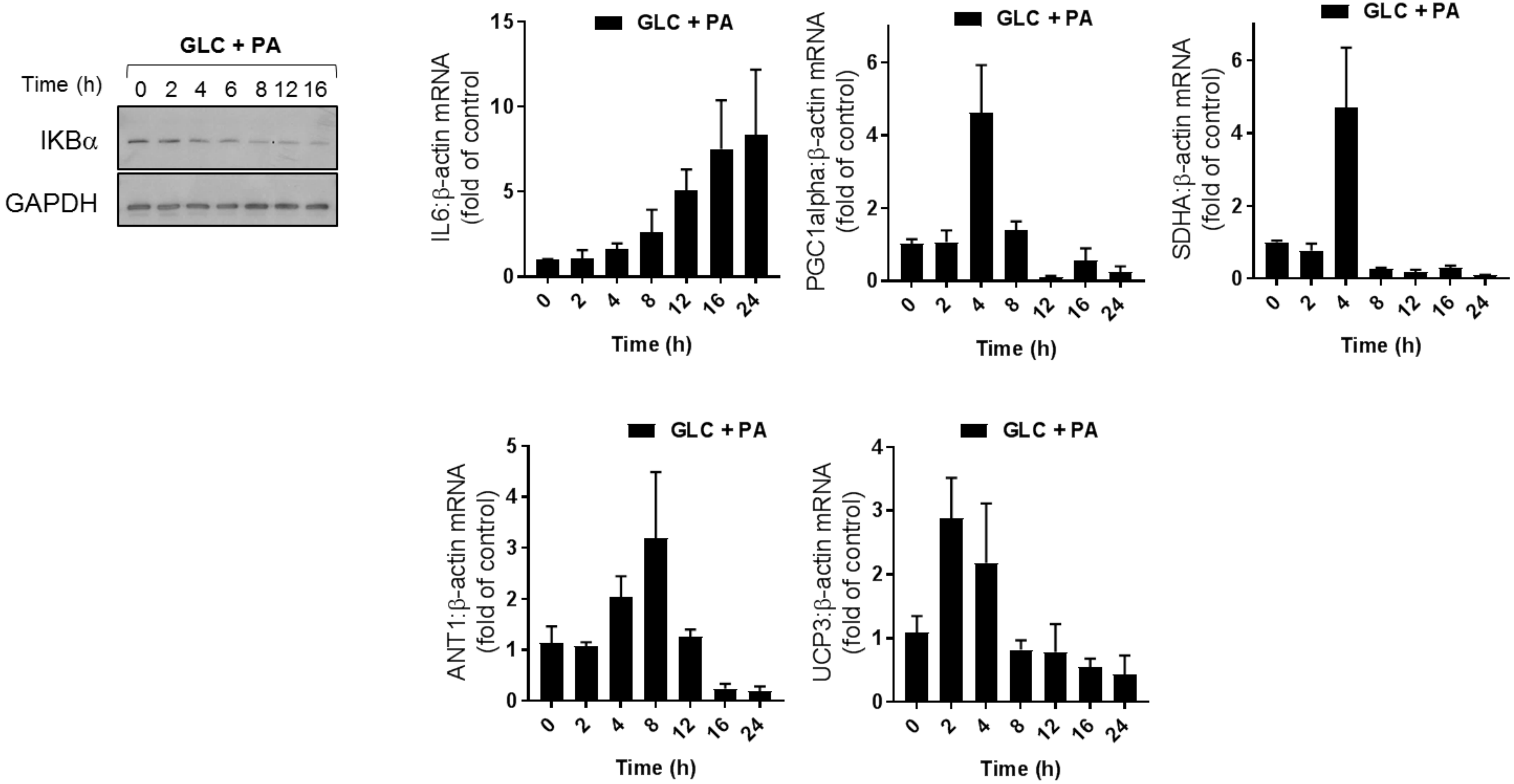

GLC + PA
Time (h)
0 2 4 6 8 12 16
IKBα
GAPDH
GLC + PA
IL6:β-actin mRNA (fold of control)
15
10
5
0
0 2 4 8 12 16 24
Time (h)
GLC + PA
PGC1alpha:β-actin mRNA (fold of control)
6
4
2
0
0 2 4 8 12 16 24
Time (h)
GLC + PA
SDHA:β-actin mRNA (fold of control)
6
4
2
0
0 2 4 8 12 16 24
Time (h)
GLC + PA
ANT1:β-actin mRNA (fold of control)
5
4
3
2
1
0
0 2 4 8 12 16 24
Time (h)
GLC + PA
UCP3:β-actin mRNA (fold of control)
4
3
2
1
0
0 2 4 8 12 16 24
Time (h)

FIGURE S6
